# Omics-based profiling and therapeutic potential of natural components in pan-Shennongjia medicinal herbs

**DOI:** 10.1186/s13020-025-01208-9

**Published:** 2025-10-16

**Authors:** Jun Song, Chong Yuan, Fei Wang, Di Lei, Xufang Tian, Lan Yang, Zhong Li, Xinxin Yi, Shi Chen, Yuling Zeng, Wei Li, Rui Deng, Qi Tao, Lingli Zhang, Yuting Wang, Ye He, Qingyu Reng, Xuan Wen, Yufeng Tan, Chi Song, Wei Chen, Wenke Xiao, Liang Leng, Sanyin Zhang, Junbo Gou, Lin Zhang, Kun Yu, Zenggen Liu, Denglang Zou, Zhaohua Shi, Liuling Pei, Zhigang Hu, Yifei Liu

**Affiliations:** 1 Hubei Shizhen Laboratory, Wuhan, 430061 China; 2 College of Pharmacy, Hubei University of Chinese Medicine, Wuhan, 430065 China; 3 Innovative Institute of Chinese Medicine and Pharmacy, Chengdu University of Traditional Chinese Medicine, Chengdu, 611137 China; 4 Institute of Herbgenomics, Chengdu University of Traditional Chinese Medicine, Chengdu, 611137 China; 5 School of Basic Medical Sciences, Hubei University of Chinese Medicine, Wuhan, 430065 China

**Keywords:** Multi-omics, Medicinal herbs, Natural products, Shennongjia, Therapeutic potential, Target mining, Artificial intelligence

## Abstract

The pan-Shennongjia region represents a globally significant biodiversity hotspot characterized by high species diversity and endemism. While its rich medicinal resources have long been recognized, the systematic characterization of their natural components and therapeutic potential remains underexplored. Here, we integrated 405 representative biological species from the pan-Shennongjia region, corresponding to 323 traditional Chinese medicine materials, into a Chinese genus-species level phylogenetic tree. We identified clade-specific species enrichments at the family level within this region. Notably, case studies of *Chrysanthemum indicum* var. *aromaticum* and *Dendrobium flexicaule* and *Chrysanthemum indicum* var. *aromaticum* revealed specificized accumulations of polysaccharides and volatile terpenoids, respectively, suggesting an environmentally-driven adaptive diversification of metabolomic profiles in pan-Shennongjia herbs. To comprehensively characterize this, we constructed a pan-Shennongjia Herbs Multi-Omics Components (SHMC) database, integrating over 20 million diverse omics-based molecules including small RNAs, small peptides, secondary metabolites, and carbohydrates. Analysis of the components distribution patterns across species revealed phylogenetic selectivity. To validate the accuracy of the annotated components, we systematically analyzed secondary metabolites and small RNAs in *Coptis chinensis*, and small peptides in *Scolopendra subspinipes mutilans* based on additional transcriptomic and metabolomic data, and further evaluated their therapeutic potential. This study establishes a crucial foundation for the conservation and sustainable utilization of pan-Shennongjia’s medicinal resources, offering the first regional-scale omics-based component database for mining valuable natural products.

**Supplementary Information:**

The online version contains supplementary material available at 10.1186/s13020-025-01208-9.

## Introduction

The pan-Shennongjia region, comprising Shennongjia and its adjacent mountain systems, represents a globally important mid-latitude biodiversity hotspot and a distribution center for China’s endemic seed plants, characterized by exceptional species diversity and remarkable endemism [1]. This region harbors over 3,000 medicinal taxa, including a large number of rare and unique species named after Shennongjia (e.g., *Chrysanthemum indicum* var. *aromaticum*, (Shennong Xiangju), *Epimedium shennongjiaensis*, and *Clematis shenlungchiaensis*), exhibiting great potential for diverse therapeutic applications. In traditional Chinese medicine culture, legend holds that Emperor Yan Shennong gathered herbs and tasted hundreds of plants mainly in the pan-Shennongjia region, encountering seventy poisons in a single day—a vivid reflection of the remarkable diversity of regional herbal medicine resources. Moreover, the Divine Farmer’s Materia Medica (Shennong Bencao Jing), China’s oldest traditional Chinese medicine compendium, also documents a substantial number of medicinal herb resources from this region. Nevertheless, the majority of medicinal resources in pan-Shennongjia region have not been subjected to thorough investigation. The natural components and therapeutic potentials of most endemic medicinal taxa remain largely unexplored, posing significant challenges to their effective preservation and sustainable utilization.

Numerous natural products synthesized by living organisms are being utilized as a primary source for food, shelter, and curative remedies. Especially, many secondary metabolites like terpenoids, alkaloids and phenolics are significant therapeutic agents beneficial for human health. Advanced researches have further expanded the content of natural products to encompass multi-level genetic or gene-encoded molecular variants, including small RNAs (sRNAs) and small peptides as medicinal agents. In traditional Chinese medicine decoction, sRNAs and proteinaceous components (including bioactive peptides) are widely identified and could enter into human being bodies via oral administration with potentially therapeutic effects [2, 3]. A study discovered that thousands of unique sRNAs derived from traditional Chinese medicine herbs can enter mammalian cells and tissues [4]. Similarly, a multitude of plant peptides demonstrates anti-microbial, anti-oxidant, anti-carcinogenic, anti-diabetic, and neuro-regulating properties [5, 6]. Therefore, a systematic characterization and comprehensive understanding of the natural components in multi-dimensional data is critically needed.

Existing databases cataloging natural components predominantly originate from a range of common species resources without distinctly regional characteristics. These databases aim to feature the representative of the most extensively studied and numerically dominant classes. For instance, the COCONUT database (https://coconut.naturalproducts.net/) integrates publicly available data, compiling near 700,000 natural products, making it one of the largest publicly accessible natural product repositories. Other notable specialized databases include Phytochemdb [7] and MetaDb [8], which focus on plant-derived metabolites, along with NP Atlas [9], dedicated to bacterial and fungal natural products. The recently introduced Gene-encoded Natural Diverse Components Repository (GNDC) [10] (https://cbcb.cdutcm.edu.cn/gndc/) systematically organizes natural products as either direct or indirect gene-encoded compounds. In addition to include the publicly available secondary metabolites and carbohydrates, this database incorporates sRNAs and small peptides primarily derived from annotated nuclear and organellar genome data of species documented in eight global pharmacopoeias. However, given the unique biodiversity and endemism in regions like pan-Shennongjia, substantial gaps remain in data coverage for endemic medicinal species, including those omics-based annotating data and experimentally confirmed natural components.

Here, we systematically investigated the phylogenetic placement of the representative pan-Shennongjia medicinal taxa within the tree of life, and characterized the specialized natural components in selected species. Based on multi-omics data, we further constructed a pan-Shennongjia Herbs Multi-Omics Components (SHMC) database to integrate diverse components of sRNAs, small peptides, secondary metabolites and carbohydrates for pan-Shennongjia medicinal herbs, and include an artificial intelligence (AI)-assisted dialogue system for data mining. Compared to COCONUT, SHMC not only collects natural components but, more importantly, establishes explicit correlations between species and their constituents. It also incorporates a substantial number of natural non-secondary metabolites, significantly expanding the diversity of natural components. In contrast to GNDC, SHMC adopts a more focused approach, emphasizing species with distinct regional characteristics. Furthermore, it integrates multi-omics data for component discovery, enhancing the accuracy and reliability of the dataset. Given the remarkable medicinal value of pan-Shennongjia regional-specific herbal resources, we conducted experimental validations of annotated components (e.g., sRNAs and small peptides) and in silico analyses to assess the therapeutic potential of the components from selected pan-Shennongjia species. This work lays a foundational framework for future conservation and sustainable utilization of pan-Shennongjia medicinal herbs and provides a reference for guiding the discovery of novel bioactive compounds from region-specific biological resources.

## Materials and methods

### Phylogenetic analysis and visualization

Two phylogenetic trees were constructed using the U.PhyloMaker package in R (v4.3.1) [11], which requires three input files: a species list, a megatree file, and a genus–family association list. The species list was compiled and standardized based on multiple sources: (1) the Flora of China (FoC) list, adapted from the Chinese genus-species level Tree of Life [12], which includes all families and over 94% of angiosperm genera listed in the FoC, following the APG IV [13] classification system; (2) all angiosperm species documented in the 2025 edition of the Pharmacopoeia of the People’s Republic of China (ChP); and (3) the pan-Shennongjia medicinal taxa to be used for SHMC database construction. We selected these pan-Shennongjia herbs mainly based on their medicinal representativeness in both traditional Chinese medicine and regional folk medicinal practices. Species names were standardized using The Plant List database (https://www.theplantlist.org/). The GBOTB.extended.TPL.tre megatree provided by U.PhyloMaker was used as the phylogenetic backbone, and the corresponding genus–family list was also obtained from this resource. The two phylogenies were pruned from the megatree, and missing medicinal plant species were incorporated using Scenario 3 of U.PhyloMaker. one of three available options and the most commonly used methods for incorporating taxa into the megatree [11]. Non-vascular plants and animal species were added as polytomies at the root of the tree using the bind.tree function from the ape package in R. These branches were included solely for visualization purposes and were excluded from subsequent phylogenetic analyses, as they do not contribute phylogenetic information. Tree visualization was performed using iTOL (Interactive Tree of Life) [14].

To identify plant families that are preferentially present in the pan-Shennongjia region compared to the ChP, an enriched family analysis was performed based on family-level usage frequency data. Specifically, the 2 × 2 contingency tables were constructed for each plant family, using the number of species belonging to that family in the pan-Shennongjia species database and in the ChP, respectively. Fisher’s exact test (one-sided, alternative ="greater") was then applied to assess whether a particular family was significantly overrepresented in the pan-Shennongjia herbs. The total number of species in each source was used to compute the expected distribution under the null hypothesis of no preferential use. A *P* < 0.05 was considered to indicate significant enrichment. Statistical analysis was performed using the fisher_exact function in the scipy.stats package in Python [15].

Phylogenetic relationships between pan-Shennongjia herbs and species listed in the ChP were analyzed using the phylocomr package in R. The functions comdist and comdistnt were used to calculate mean phylogenetic distance (MPD) and mean nearest taxon distance (MNTD), which reflect deeper and more terminal levels of phylogenetic structure, respectively. Standardized effect sizes—Net Relatedness Index (NRI) for MPD and Nearest Taxon Index (NTI) for MNTD—were calculated to assess phylogenetic overdispersion or clustering.

### Characterizing volatile terpenoids in *Chrysanthemum indicum* var. *aromaticum*

*C. indicum* var. *aromaticum* samples were collected from the Shennongjia Forestry District, Hubei Province, China. *C. indicum* samples were collected from Dawu County, Hubei Province, China. Flower samples from both species were harvested during their respective flowering periods (*C. indicum* var. *aromaticum*: September 2022; *C. indicum*: November 2022). For subsequent experiment, six biological replicates per species were prepared. Each replicate consisted of a composite sample created by thoroughly mixing flowers collected from ten individual plants. Given the distinctive aromatic profile of *C. indicum* var. *aromaticum*, we analyzed its volatile organic compounds (VOCs) using an optimized headspace solid-phase microextraction coupled with gas chromatography-tandem mass spectrometry (HS–SPME–GC–MS/MS) method based on a widely targeted volatomics (WTV) approach [16, 17]. Briefly, 500 mg of each sample was thoroughly mixed with 10 μL of 3-Hexanone-2,2,4,4-d4 (50 μg/mL) and a saturated NaCl solution. The resulting mixture was immediately transferred into a 20 mL headspace vial. VOCs were then extracted using a 120 μm DVB/CWR/PDMS fiber (Agilent), followed by thermal desorption in the GC inlet prior to analysis. Separation was achieved on a DB-5MS capillary column with ultra-high purity helium as carrier gas. MS detection employed electron impact ionization (70 eV) with ion source, quadrupole. Data acquisition in selected ion monitoring mode utilized MassHunter software aligned with GB 23200.8–2016 standards. Compound identification required mass spectral matches (NIST database, match factor ≥ 70) and confirmation through characteristic fragment ions. Relative VOCs concentrations (Ci, μg/g) were calculated using the formula: Ci = (Ii × Vs × Cs)/(Is × M), where Vs (μL) and Cs (μg/mL) denote internal standard volume/concentration, M (g) is sample mass, and Ii/Is represent analyte/internal standard peak areas.

To investigate the biosynthesis of the identified volatile terpenoids, we conducted differential metabolomic analysis using the MetaboAnalystR package [18] (v1.0.1) in R based on the available transcriptomic and metabolomic data of *C. indicum* var. *aromaticum*. To construct the TPS-terpenoid co-expression network, transcriptomic data from our previously published study (Accession number: PRJCA029413) and metabolomic data obtained in this study were integrated. Pearson correlation coefficients between the abundance levels of 99 upregulated terpenoids and the expression levels of 112 terpene synthase (TPS) genes were calculated using the psych package (version 1.9.12.31) within the R environment. The filtered TPS-terpenoid correlation pairs were visualized using Cytoscape software [19] (v3.9.1). For functional validation of selected TPS genes, candidate genes exhibiting strong correlations (R > 0.9, *P* < 0.05) with the upregulated terpenoids within the co-expression network were stringently screened. Full-length coding sequences of these candidates were PCR-amplified using gene-specific primers (Table S1) and ligated into the His-tagged pET-28a (+) vector using the ClonExpress® II One Step Cloning Kit (Vazyme Biotech). Subsequent prokaryotic expression and in vitro enzymatic assays were performed following a previously described protocol [20]. The identified enzymatic products were validated by matching their retention times to authentic standards and cross-referencing with the NIST mass spectral database.

### The extraction and antioxidant activity evaluation of *Dendrobium* polysaccharides

To investigate the specific accumulation of polysaccharides in *Dendrobium flexicaule*, a precisely weighed amount of herbal powder was extracted twice with water at 60 °C for 1.5 h. The extraction methods for *D. officinale* and *D. huoshanense* were the same. *D. flexicaule* was collected from the Shennongjia Forestry District, Hubei Province, China, *D. officinale* was obtained from the Hefei, Anhui Province, China and *D. huoshanense* was obtained from the Huoshan County, Anhui Province, China. The combined extracts were precipitated with anhydrous ethanol, and the resulting precipitate was dried and weighed to determine the polysaccharide yield [21]. The antioxidant capacity was assessed by using ABTS and FRAP assays [22, 23]. ABTS⁺ clearance rate was determined by incubating samples with ABTS working solution in 96-well plates at room temperature in the dark for 5 min. Absorbance was read at 734 nm, and clearance rate was calculated using the formula: [1 − (A sample – A blank)/A blank] × 100%. For FRAP analysis, FRAP reagent and FeSO₄ standards (0.1–1.0 mmol/L) were incubated (37 °C, 30 min), and absorbance was measured at 593 nm to generate a standard curve. The antioxidant capacity of samples was then determined by measuring the absorbance difference (A sample–A blank) under the same conditions and comparing to the standard curve.

### SHMC database construction

#### Data acquisition and processing

The pan-Shennongjia species employed for phylogenetic analysis were used for database construction. The fundamental species information includes the scientific nomenclature (Latin name), habitat distribution, and therapeutic applications in traditional Chinese medicine, with references mainly from the ChP, FoC, peer-reviewed publications and relevant documentary sources. We collected multi-omics data associated natural components of these species with alternative approaches. For secondary metabolites, experimentally derived metabolomics data, literature resources, and multiple open-source database, including GNDC, TCMSP [24] (https://www.91tcmsp.com/), TCMBank [25] (https://tcmbank.cn/) and HERB2.0 [26] (http://herb.ac.cn/v2) were integrated. All secondary metabolites underwent a two-stage quality control process, including initial manual check followed by computational deduplication to eliminate redundant entries. Secondary metabolites classification was automated using NPClassifier [27].

The species genome data collected from the National Center for Biotechnology Information (NCBI) (https://www.ncbi.nlm.nih.gov) and 1 K Medicinal Plant Genome Database [28] (http://www.herbgenome.com) were employed for genome-wide prediction of small peptides using a six-frame translation approach implemented in EMBOSS [29] and ORFIPY [30]. Filtering criteria were applied to retain sequences ranging from 5 to 75 amino acids in length. Small peptides were functionally analyzed against established protein function and pathways. KEGG pathway annotations were assigned via HMMscan [31] to identify associated metabolic and signaling pathways. Functional classification was enhanced through BLASTp homology searches against the UniProt. Physicochemical properties (molecular weight, isoelectric point and amino acid numbers) were predicted using the ProtParam [32] module within Bio.SeqUtils. Anti-inflammatory potential was assessed with PepNet [33], while anti-microbial activity was predicted using iAMPCN [34].

The small RNAs data consists of four types of miRNA, rRNA, tRNA, and snRNA. The Rfam database [35] (v14.10) alongside INFERNAL software [36] (v1.1.4) were used for predicting ncRNAs, which were further refined to extract snRNAs and miRNAs. tRNAscan-SE [37] (v2.0.12) and rRNAmmer [38] (v1.2) was employed for tRNA and rRNA prediction, respectively. miRDeep2 [39] (v2.0.1.2) software was also used for additional miRNA prediction based on genome sequences and miRbase [40] (v22.1) database.

The carbohydrates data were compiled from GlyTouCan (https://glycosmos.org/download) [41]. Corresponding information includes species, SMILES, IUPAC name, CID, molecular weight, and molecular formula. The duplicate carbohydrate entries were identified and removed by comparing their SMILES and species information to ensure data consistency.

#### Target prediction of secondary metabolites

The ADMET properties of secondary metabolites were evaluated using ADMET-AI [42], a predictive tool based on the Chemprop-RDKit deep learning framework. This framework integrates a directed message-passing neural network (D-MPNN) with 200 RDKit-derived molecular descriptors. Molecular feature vectors were generated by aggregating atomic hidden states, enabling accurate prediction of absorption, distribution, metabolism, excretion, and toxicity (ADMET) profiles. These predictions provided critical insights into the drug-likeness of secondary metabolites. Finally, two target prediction frameworks based on Morgan circular fingerprints and machine learning paradigms (e.g., random forest, neural networks) were instantiated to establish a secondary metabolite target deconvolution workflow.

#### AI-assisted data mining module

We developed an AI dialogue system by fine-tuning the DeepSeek 671B R1 model [43] to assist data mining tasks. The system integrated technologies including NL2SQL for precisely translation of natural language into database query commands for efficient data retrieval, RAG (Retrieval-Augmented Generation) technology to enhance herbal multi-omics components data understanding, and intelligent agent development technology to simulate expert logical thinking for problem analysis and resolution. Additionally, we implemented vector database indexing for efficient herbal medicine data storage and retrieval.

#### Website development

The SHMC database website platform (https://shmc.hbucm.edu.cn/) was developed using the JFinal framework and leverages PostgreSQL as the database backend on Linux. JFinal’s Object-Relational Mapping (ORM) was employed to facilitate secure and efficient data management. The frontend is built with HTML5 and Vue 3, with interactive features implemented through JavaScript and jQuery. To optimize performance, PostgreSQL was deployed as a cluster. Additionally, the platform integrated robust security measures, including HTTP interception, data encryption, and Cross-Site Request Forgery (CSRF) protection, ensuring the integrity and confidentiality of biological data.

### Targets screening and enrichment analysis of alkaloids in *Coptis chinensis*

The *C. chinensis* alkaloids in the SHMC database were batch-processed using their SMILES notations in ADMET-AI (https://admet.ai.greenstonebio.com). Screening was performed with the following thresholds: molecular weight < 500 Da, no more than 5 hydrogen bond donors, ≤ 10 hydrogen bond acceptors, and a lipid-water partition coefficient (log P) value ≤ 5. The targets of the screened components were then predicted using the SHMC tools and merged to remove duplicates. The predicted targets were imported into Metascape (https://metascape.org/gp/index.html#/main/step1) for KEGG and GO enrichment analyses. Results were filtered by significance (*P* < 0.05), and the top 25 enriched pathways based on the correlation ships were selected and consolidated, and their corresponding components were identified. Finally, a “component-target-pathway” network was constructed and visualized using Cytoscape (v3.8.1) software, and molecular docking was performed on the top-ranked compounds and targets using Autodock [44] Vina software.

### Identification and target gene prediction of miRNAs in *C. chinensis*

Four years old *C. chinensis* plants were collected from Lichuan, Hubei province. Total RNA was extracted from five tissues (leaf, rhizome, fibrous root, stem, and fruit) using TRIzol reagent. Small RNA libraries were prepared with the KC-DigitalTM small RNA Library Prep Kit and sequenced on an Illumina Hiseq X-10 platform after quality filtering. The TBtools software [45] (vJRE1.6) was utilized to remove adapter sequences. Reads were then merged and deduplicated, and miRNAs were predicted using the miRDeep2 software based on the *C. chinensis* reference genome [46] and the miRBase database. The predicted miRNAs were further cross-referenced with SHMC database using Bowtie2 [47]. The complementary pairing patterns between the SHMC overlapping miRNAs and human mRNAs were analyzed using the RNAhybrid [48] software (v2.1.2) to predict potential miRNA binding sites, followed by KEGG pathway enrichment analysis of targets using clusterProfiler package in R. The miRNA-target gene-disease pathways were visualized using Cytoscape software [49].

### Identification and functional prediction of small peptides in *Scolopendra subspinipes mutilans*

We predicted small peptides in *S. subspinipes mutilans* used both transcriptomic and genomic data. For transcriptome sequencing, both male and female *S. subspinipes mutilans* samples were collected from Suizhou, Hubei Province, China. Total RNA was extracted to construct libraries using MGIEasy RNA Library Prep Kit and sequenced on BGI high-throughput sequencing platform DNBSEQ. Clean reads were aligned to the *S. subspinipes mutilans* reference genome [50], with transcripts assembled using StringTie [51] (v3.0.0) and coding sequences identified via TransDecoder [52] (v5.7.1). Transcriptomic and genomic data derived peptides were compared using BLAST (e-value 1e^−5^), retaining hits with qcovs ≥ 80% in BLASTp as validated peptides. The position of genomic feature region was extracted using bedtools [53] (v2.31.1) and gffread [54] (v0.12.7), including exon, intron, TSSup2k, TESdown2k, and the rest named non-promoter-terminal region (Nptr). After removing duplication, bedtools intersect parameter was used to mine the validated peptides, which position coordinates lie entirely in these genomic feature regions. Visualization of data was performed using R.

## Results

### Diversity and phylogenetic representative of pan-Shennongjia medicinal herbs

**Fig. 1:**
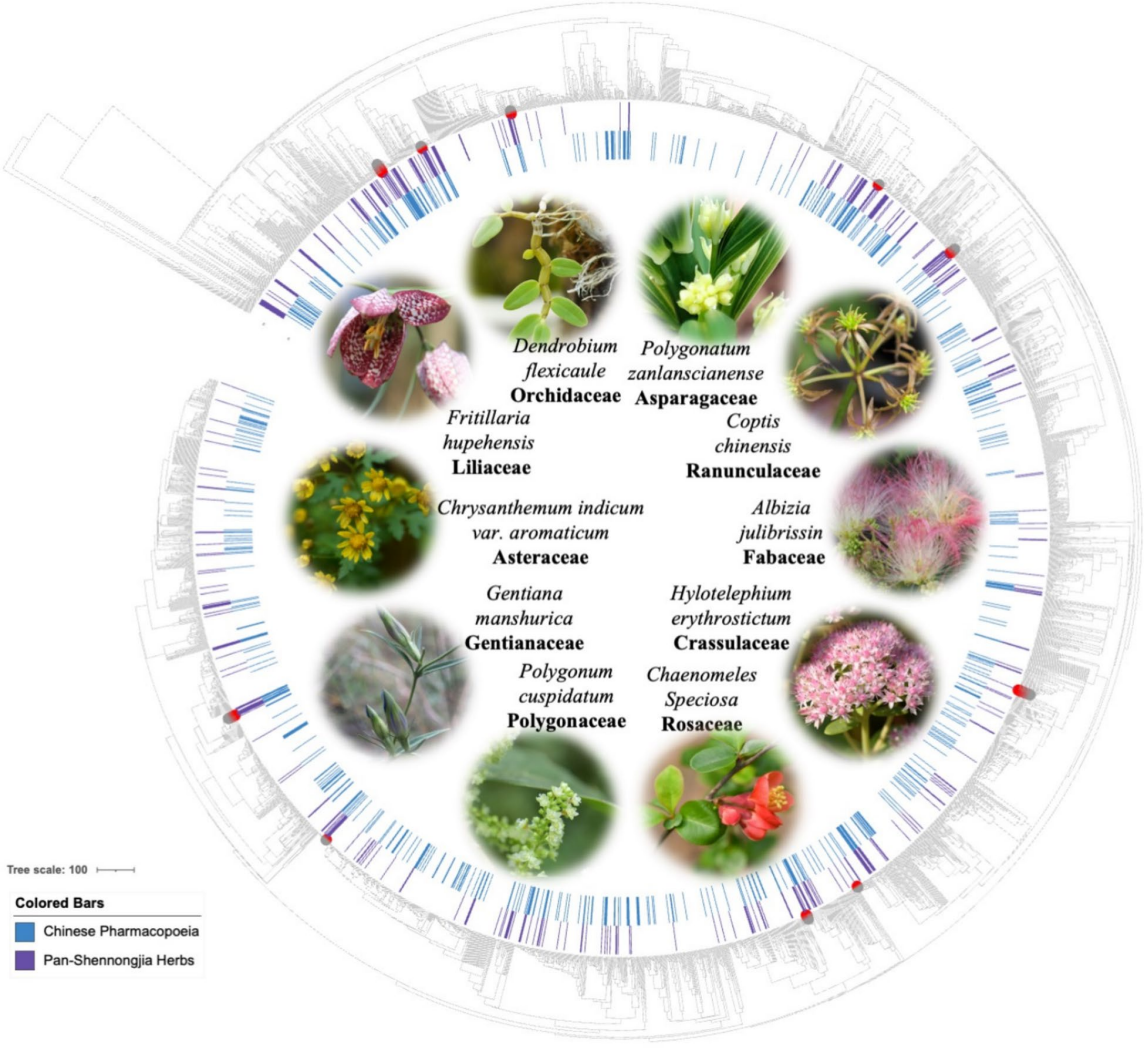
Phylogenetic structure and represented species and family of pan-Shennongjia herbs. Phylogenetic tree of angiosperm species from the pan-Shennongjia region and ChP constructed against the FoC species pool. Inner colored rings indicate species included in ChP (purple) and pan-Shennongjia herbs (blue). Representative species from the pan-Shennongjia region, belonging to the top 10 most frequently used plant families, were highlighted in the central area

Based on species distribution records from the ChP, FoC, and the Divine Farmer’s Materia Medica, we identified 405 representative medicinal species belonging to 104 families in the pan-Shennongjia region. For plants, the most frequently used families—ranked by species count—were Asteraceae, Ranunculaceae, Polygonaceae, Rosaceae, Orchidaceae, Fabaceae, Liliaceae, Crassulaceae, Asparagaceae, and Gentianaceae. Representative species from each of these families were also shown in Fig. 1. These families reflected both taxonomic diversity and a wide range of phytochemical profiles commonly associated with traditional herbal use. However, frequency alone does not necessarily reflect preferential selection [55]. To identify plant families that are disproportionately represented in pan-Shennongjia herbal knowledge compared to the ChP, we performed a family-level enrichment analysis using Fisher’s exact test. Several families were found to be significantly enriched (*P* < 0.05), including Crassulaceae, Polygonaceae, Orchidaceae. This suggested that the pan-Shennongjia herbs emphasizes different botanical lineages compared to the nationally standardized record. These enriched families likely reflected regionally adapted ethnomedical practices and local ecological availability [56].

The phylogenetic structure of pan-Shennongjia herbal angiosperm species was assessed in comparison with those recorded in the ChP. Phylogenetic analysis revealed no significant structure at deeper taxonomic levels (MPD-based Net Relatedness Index, NRI: not significant, Table S2), but showed strong phylogenetic overdispersion near the tips (MNTD-based Nearest Taxon Index, NTI: over dispersed, *P* < 0.001, Table S2). This pattern indicated that pan-Shennongjia herbs and species recorded in the ChP tend to include distantly related taxa at finer phylogenetic scales. Such tip-level overdispersion suggested that pan-Shennongjia herbs reflect a broader or distinct selection strategy compared to the ChP (Fig. 1), possibly shaped by regional knowledge systems or ecological conditions [57, 58].

### Component specificity of *Chrysanthemum indicum* var. *aromaticum*

**Fig. 2:**
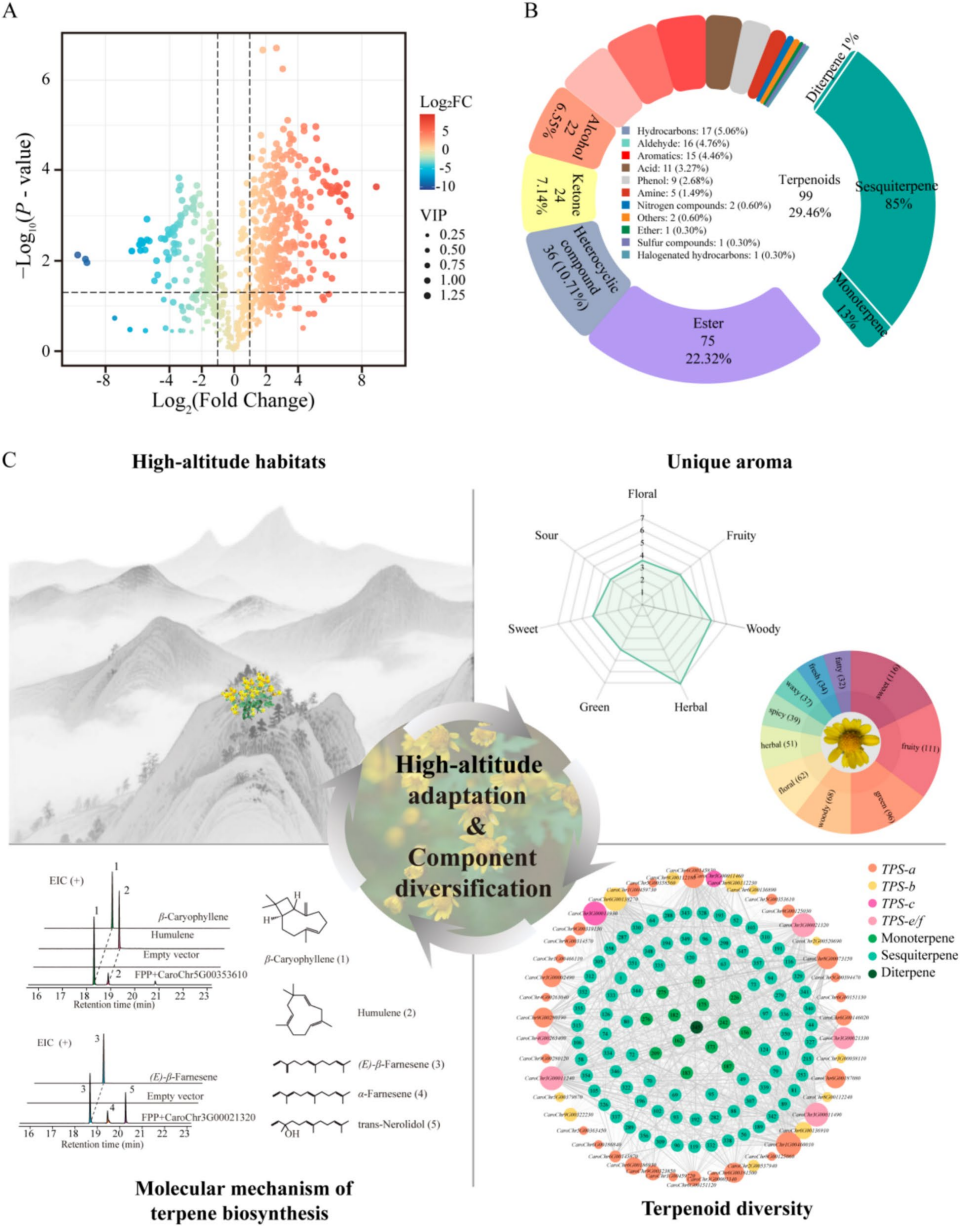
Characterization and environment mediated biosynthesis of volatile terpenoids in *Chrysanthemum indicum* var. *aromaticum*. **A** Volcano plots of differential volatile metabolites between *C. indicum* var. *aromaticum* and *C. indicum*. **B** Taxonomic classification of upregulated volatile metabolites in *C. indicum* var. *aromaticum*. **C** Environment—adaptation molecular mechanism in the case of *C. indicum* var. *aromaticum*. The component information associated with each identification number in the TPS-terpenoid co-expression network is detailed in Table S3

The Asteraceae family represents one of the highest levels of diversity and specificity among medicinal plant families in the pan-Shennongjia region (Fig. 1). One particular note is *C. indicum* var. *aromaticum*, an Asteraceae plant endemic to the high-altitude areas (> 2000 m) of pan-Shennongjia region. While *C. indicum* var. *aromaticum* has been taxonomically identified as a variant of *C. indicum*, it exhibits a characteristic aroma profiling distinguished from *C. indicum*, which may be attributed to the unique high-altitude adaptation. To elucidate its unique aroma composition, we conducted widely-targeted volatile metabolomics on both *C. indicum* var. *aromaticum* and *C. indicum*. Among the 958 identified metabolites, 336 compounds exhibited species-specific hyperaccumulation in *C. indicum* var. *aromaticum* (Fig. 2A). Terpenoids were the dominant hyperaccumulated metabolites (99 compounds, 29.5% of total), in particular sesquiterpenes (85%, n = 84) and monoterpenes (13%, n = 13) (Fig. 2B, Table S3).

Terpenoid biosynthesis in plants occurs mainly through both the mevalonate (MVA) and methylerythritol phosphate (MEP) pathways, mediated by the key enzymatic activity of TPS [20]. To investigate the molecular mechanisms underlying the aromatic profile of *C. indicum* var. *aromaticum*, we identified potential biosynthetic enzymes for the 99 upregulated terpenoids by integrating transcriptomic and metabolomic datasets. A TPS-terpenoid co-expression network was constructed to delineate the potential relationships between TPS and terpenoids (Fig. 2C). Functional characterization via prokaryotic expression of two selected TPS revealed their catalytic versatility, producing five aroma-active terpenoids detected in *C. indicum* var. *aromaticum* (Fig. 2C). Notably, three of these compounds exhibited species-specific hyperaccumulation, indicating *TPS* gene functional diversification constitute the intrinsic molecular basis for its distinctive aroma profile. To date, 26 terpenoids showing specific accumulation in *C. indicum* var. *aromaticum* have been biochemically validated as products of specialized TPS (Table S4). This compelling evidence established that TPS diversification—both in gene copy number and catalytic plasticity—directly drives the species’ characteristic component biosynthesis.

### Polysaccharide accumulation in *Dendrobium flexicaule*

**Fig. 3:**
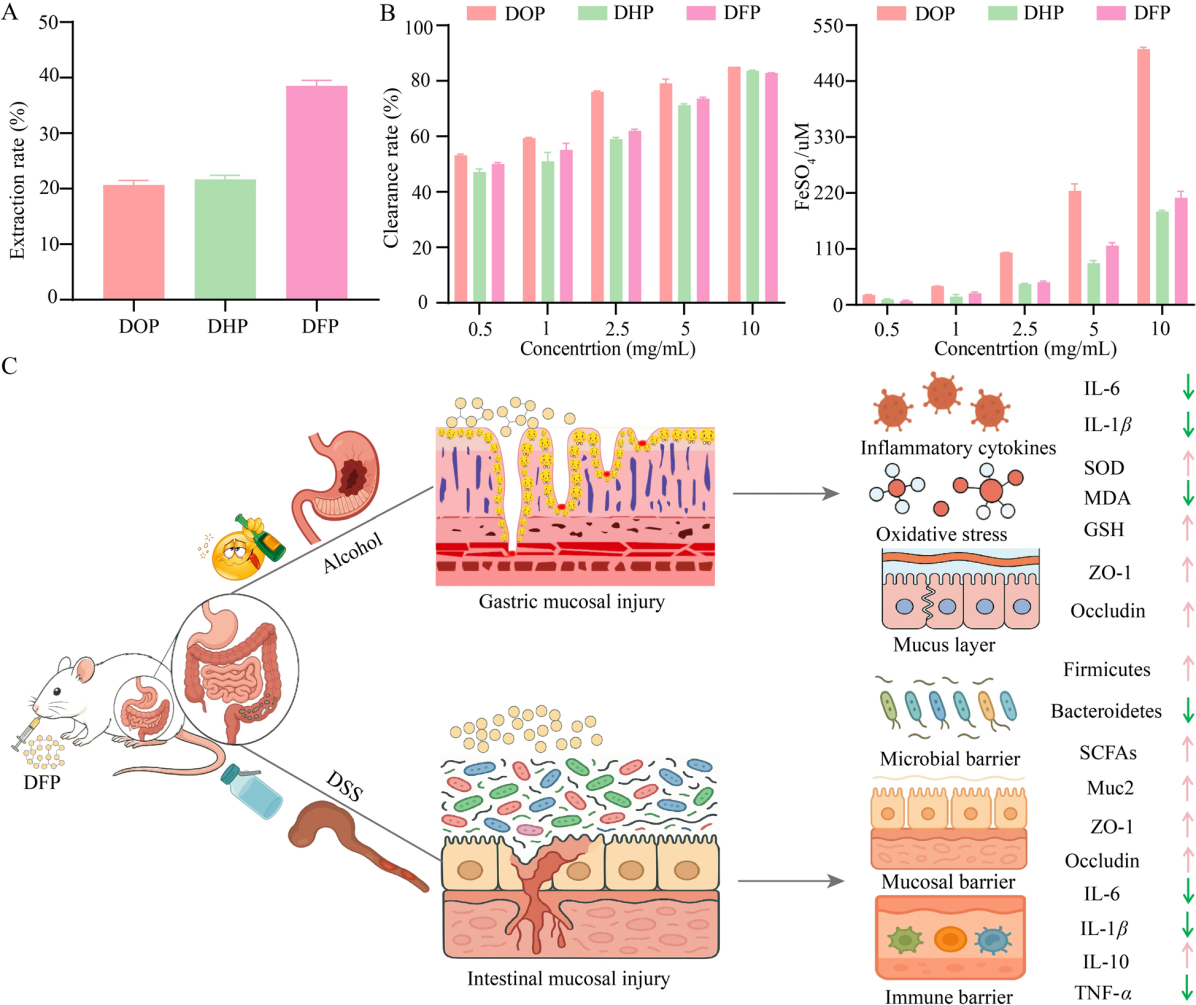
Composition and therapeutic effects analysis of *Dendrobium flexicaule* polysaccharides. **A** The extraction yields of polysaccharides from three *Dendrobium* species. **B** Evaluation of antioxidant activities of polysaccharides from three *Dendrobium* species in vitro. **C** Gastrointestinal protective effects of *D. flexicaule* polysaccharide. *DOP* *D. officinale*’s polysaccharides, *DHP* *D. huoshanense*’s polysaccharides, *DFP* *D. flexicaule*’s polysaccharides

*Dendrobium flexicaule* is a perennial herbaceous plant in the Orchidaceae family. Similar to *C. indicum* var. *aromaticum*, *D. flexicaule* is another rare and unique medicinal herb mainly distribute in pan-Shennongjia region, commonly found on damp and shady rocks at altitudes of 900 to 1500 m. Polysaccharides, as an important class of biological macromolecules, are present in many traditional Chinese medicines and are significant components of the *Dendrobium* plants. We therefore compared the crude polysaccharide extraction yields of *D. officinale*, *D. huoshanense*, and *D. flexicaule*. Both *D. officinale* and *D. huoshanense* are commonly used for clinical applications and recorded in ChP. Under the same extraction method, *D. flexicaule* had the highest crude polysaccharide extraction yield (Fig. 3A).

We further assessed in vitro antioxidant capacities of crude polysaccharides from three *Dendrobium* species using the ABTS and FRAP methods. The results showed that at a concentration of 10 mg/mL, the clearance rates for *D. flexicaule*’s polysaccharides (DFP), *D. huoshanense*’s polysaccharides (DHP), and *D. officinale*’s polysaccharides (DOP) were 82.87 ± 0.02%, 83.63 ± 0.25%, and 79.14 ± 1.51%, respectively*.* FRAP results indicated that the antioxidant capacities of DOP, DFP, and DHP at a concentration of 10 mg/mL were found to be 503.10 ± 3.23 μmol/L FeSO_4_, 210.4 ± 12.91 μmol/L FeSO_4_, and 183.51 ± 2.54 μmol/L FeSO_4_, respectively (Fig. 3B). These reflected their different antioxidant capacities. Our previous studies have confirmed that DFP has a potent protective effect on gastrointestinal mucosa, improving alcohol-induced acute gastric mucosal injury, reducing inflammatory factors and oxidative stress, and enhancing the expression of mucosal proteins in the gastric mucosal barrier [20]. We have also confirmed that DFP has an ameliorative effect on DSS-induced ulcerative colitis, functioning by modulating the intestinal flora, mucosal barrier, and immune barrier (Fig. 3C). These results indicated that *D. flexicaule* exhibited differences from *D. officinale* and *D. huoshanense*, and possessed significant therapeutic potential, consistent with the regional specificity of the pan-Shennongjia herbs.

### Construction of SHMC database

**Fig. 4:**
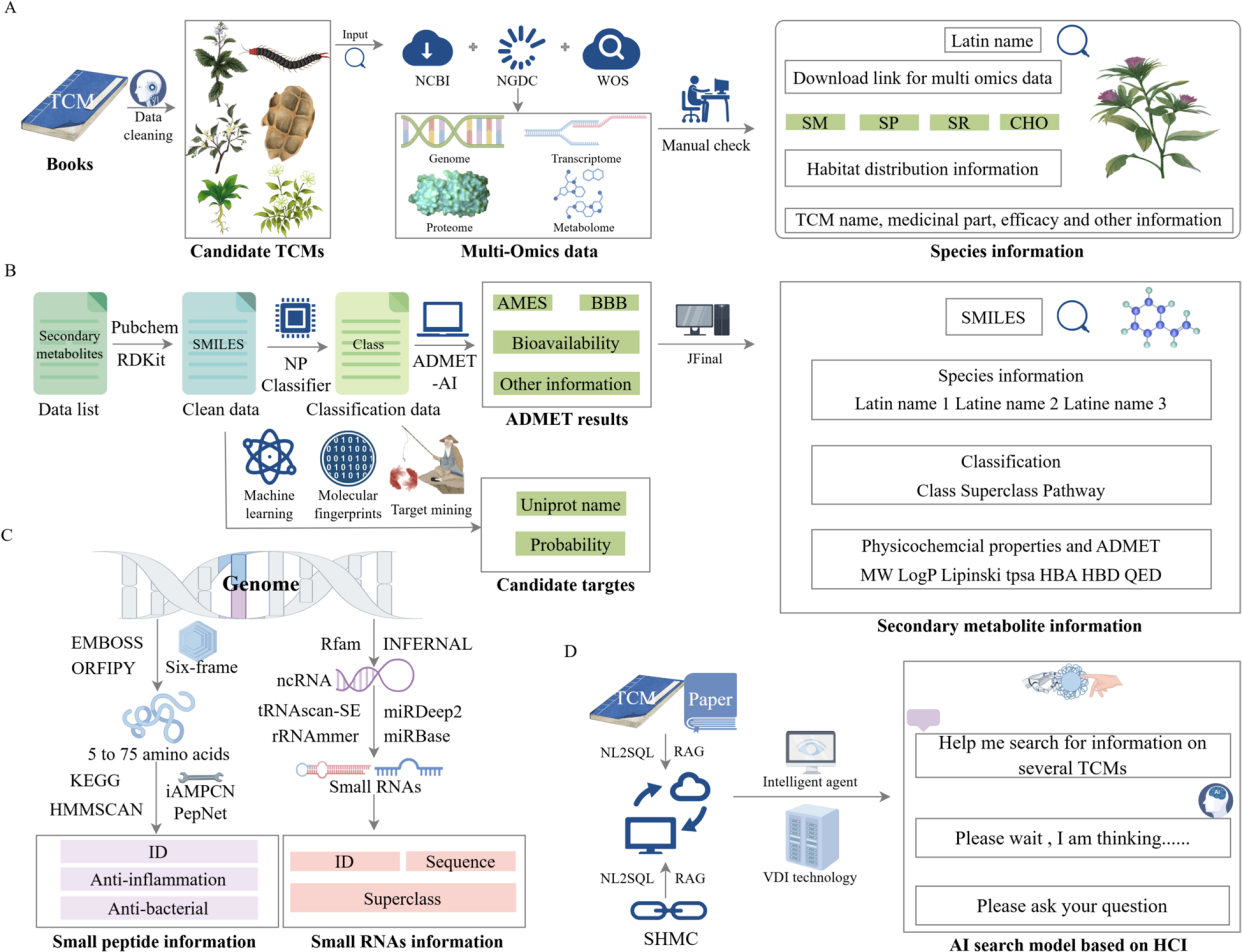
Schematic overview for the construction of the SHMC database. **A** The construction process of the 405 species information module, namely the collection, cleaning, and manual correction of their basic information and multi-omics data. **B** The construction of the secondary metabolite module, including data cleaning, classification, and ADMET prediction processes, as well as the development process of a candidate target mining tool for secondary metabolites based on machine learning and molecular fingerprints. **C** The development process of small peptide and sRNAs prediction tools based on species genome data using Six-frame and multi-database integration. **D** The development process of a pan-Shennongjia herbs intelligent query tool built on artificial intelligence models, using the SHMC database, TCM books, and published papers as data sources

To better summarize and catalog the diverse and unique natural components of pan-Shennongjia medicinal herbs like *C. indicum* var. *aromaticum* and *D. flexicaule*, and to facilitate the exploration of their therapeutic potential, we built a comprehensive database by integrating multi-omics data-based components of these species (Fig. 4). The resulting database encompassed 323 types of traditional Chinese medicine derived from 405 biological species. Information related to the habitat characters of these species and the potential medicinal properties (nature, flavor, meridian tropism) recorded in classical Chinese material medica compendium were also included. The database contained over 20 million diverse molecular components. Specifically, 850,661 sRNAs, including 21,380 miRNAs, 494,663 tRNAs, 215,985 rRNAs and 118,633 snRNAs were predicted from genome data. Additionally, 28,225,123 small peptides were annotated based on the respective genomic data. For secondary metabolites, the database incorporated 70,145 entries, along with 1410 carbohydrates, primarily sourced from public databases with supplementary data from metabolomic studies. Beyond these molecular components, the database also included 1025 raw omics data, enabling further investigations of the potential biosynthetic pathways of a targeted metabolites through advanced computational analyses.

The database also comprised specialized functional modules. A module combined the secondary metabolite ADMET profiling and target prediction modeling suite (Fig. 4B), engineered to facilitate the association of large-scale natural secondary metabolites with disease-relevant targets for the identification of potential drug candidates. Our database employed RDKit for batch-processed secondary metabolites mining, with NPClassifier assigning taxonomic classifications. ADMET-AI was applied for high-throughput profiling of 90 + ADMET descriptors, supporting hypothesis-driven analyses (e.g., prioritizing blood–brain barrier permeability in CNS drug evaluation). Users could input SMILES strings of query secondary metabolites to generate probabilistic target annotations. To optimize screening efficiency, the tool assigned confidence scores to all predicted targets, enabling users to triangulate across multiple predictive models and their associated likelihood values to derive final target hypotheses. This infrastructure provided translational insights for the prioritization of bioactive constituents in medicinal taxa and the mechanistic elucidation of their pharmacological effects.

To enhance user accessibility, we also implemented an AI-driven conversational interface (Fig. 4D) that enables intuitive exploration of medicinal species and their natural components through natural language interactions. The intelligent agent simulated expert-level logical thinking to analyze and resolve inquiries. For example, when users ask about the secondary metabolites of a specific species, the system organizes responses based on database content. Follow-up questions, such as “Which species contain a particular type of component,” can also be addressed through systematic analysis of stored data, generating corresponding species lists. Powered by database-driven deep learning, this AI system functioned as a virtual expert specializing in pan-Shennongjia herbal species and their biochemical constituents, offering professional-grade data retrieval and interpretive capabilities.

### Distribution of natural components for pan-Shennongjia herbs in SHMC

**Fig. 5:**
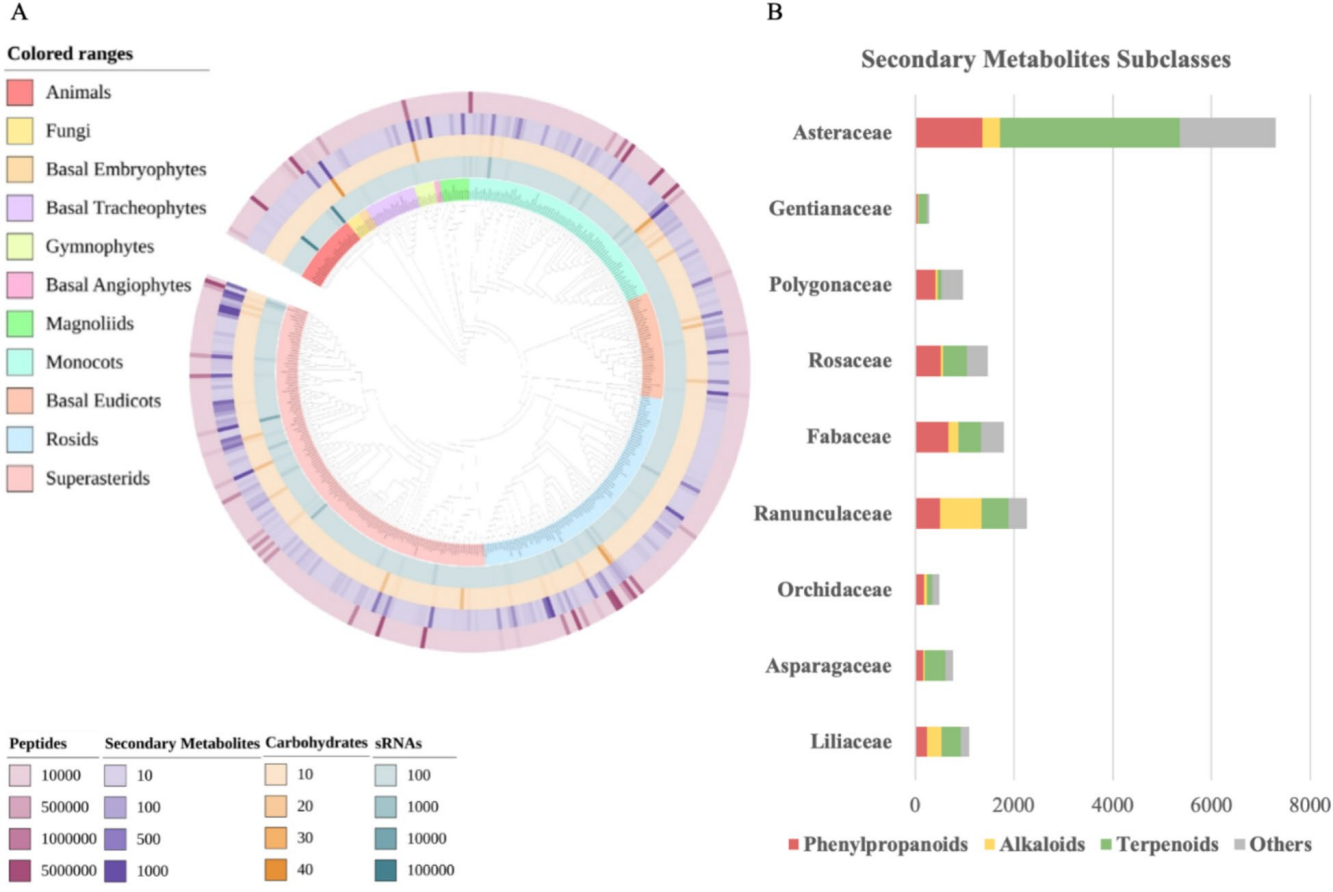
Phylogenetic visualization of natural components diversity for pan-Shennongjia herbs. **A** A comprehensive phylogeny of all species and their natural components from the pan-Shennongjia region recorded in SHMC. The innermost circle labels species names and their taxonomic classification according to APG IV. Moving outward, four rings with heatmap represent the numbers of different components categories—sRNAs, carbohydrates, secondary metabolites and small peptides—recorded in SHMC for each species. **B** The stacked columns show four subclass distribution (Phenylpropanoids, alkaloids, terpenoids and others) of secondary metabolites compiled from available species-level data in the SHMC database, aggregated by family for the top 10 most frequently used plant families in the pan-Shennongjia region, excluded crassulacean due to the insufficient data

Based on a phylogenetic tree constructed for 405 pan-Shennongjia biological species in the SHMC database, the presence and abundance of four major compound categories, including small peptides, sRNAs, carbohydrates, and secondary metabolites were exhibited (Fig. 5A). The total distribution of these components showed pronounced variation across species and taxonomic groups. Secondary metabolites were the most widely documented, with over 65% of species having at least one recorded entry. In contrast, sRNAs and carbohydrates were less prevalent, with approximately 80% and 90% of species, respectively, lacking corresponding data.

Certain genera and families showed clear patterns of compound enrichment. For example, small peptides were particularly abundant in *Moschus*, *Dendrobium*, and *Tenodera*, while secondary metabolites were enriched in plant genera such as *Chrysanthemum* and *Rheum*. Families including Asteraceae, Orchidaceae, and Ranunculaceae also stood out for their compound diversity (Fig. 5B). In contrast, some widely used families, such as Crassulaceae and Polygonaceae where 11 out of 12 and 7 out of 14 species recorded in the pan-Shennongjia region had no secondary metabolite data, appeared underrepresented at the molecular level. These observed disparities suggest that the pan-Shennongjia region not only harbors a rich diversity of traditional medicinal species, surpassing even the ChP in some families, but also holds substantial natural components diversity with strong potential for future pharmacological discovery.

### Characterization of secondary metabolites and miRNAs in *Coptis chinensis*

**Fig. 6:**
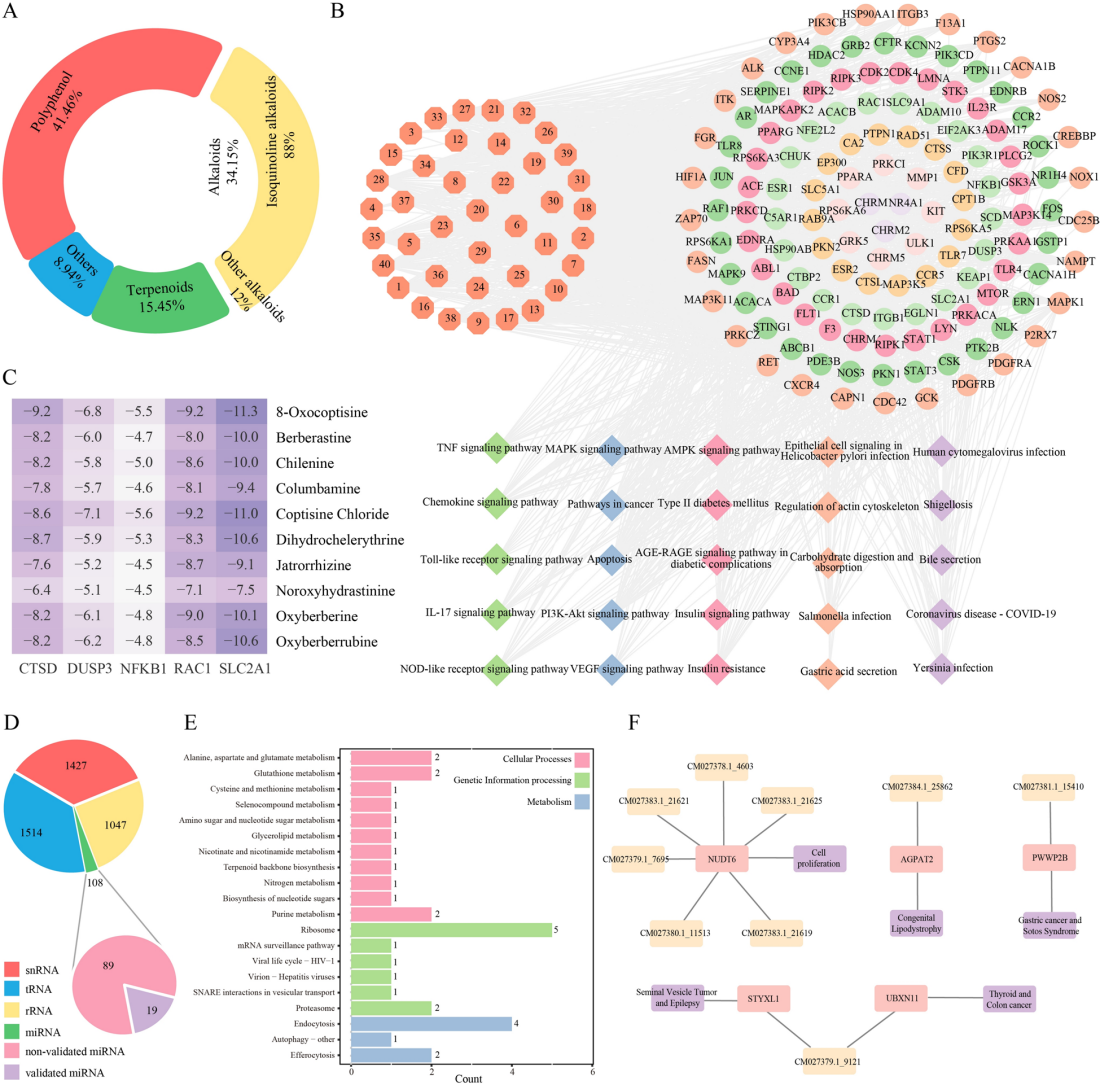
Exploration of the therapeutic potential of secondary metabolites and miRNAs in *Coptis chinensis*. **A** Classification of secondary metabolites in *C. chinensis* as collected by SHMC. **B** Component—target—pathway network, the green diamonds represent the pathways related to inflammation; blue represent pathways associated with tumor research; pink represent pathways related to diabetes; orange represent pathways associated with gastrointestinal diseases and purple represent pathways related to anti-bacterial and anti-viral activities. The component information associated with each identification number is detailed in Table S5. **C** Molecular docking energy heatmap. **D** Pie chart represents the types of sRNAs predicted by SHMC database, which are snRNAs, tRNAs, rRNAs and miRNAs, and miRNAs are divided into two categories: those verified by miRNA-seq and those not verified by miRNA-seq. **E** Bar graphs represent KEGG enrichment of SHMC predicted miRNA validated by miRNA-seq. **F** The network of disease resistance pathway of the representative miRNA target genes in KEGG enrichment results

*Coptis chinensis*, a characteristic medicinal plant, is both naturally distributed and extensively cultivated in the pan-Shennongjia region. The rhizomes of *C. chinensis* have been included many classic formulae in tradition Chinese medicine for thousands of years. Based on the SHMC database, we classified 123 *C. chinensis* secondary metabolites into alkaloids, polyphenols, terpenoids, and other components. We found that the main medicinal components of *C. chinensis*, alkaloids, accounted for 34.15% of this dataset, with 88% being isoquinoline alkaloids (Fig. 6A). Using the relevant ADMET results and targets obtained in the database of 40 alkaloids, we constructed a “component-target-pathway” network (Fig. 6B). We found the resulted signaling pathways were primarily associated with diseases such as inflammation, anti-bacterial, anti-viral, and anti-tumor activities. These diseases were closely related to the clinical applications of *C. chinensis* and its modern pharmacological effects. Studies have shown that oxyberberine [59], jatrorrhizine [60], and berberine [61] all exhibited significant effects on inflammation. Research indicateed that oxyberberine can alleviate TNBS-induced colitis in rats by modulating the Keap1/Nrf2/NF-κB pathway, which influences inflammation and oxidative stress responses, with its efficacy being stronger than that of berberine [62]. We selected the top-ranked components for docking with top targets, and both oxyberberine and jatrorrhizine demonstrated low binding energies with the targets, further validating the accuracy of the database-predicted targets (Fig. 6C).

Based on the SHMC database, we identified a total of 4,096 sRNAs in *C. chinensis*, including 1,514 tRNAs, 1,427 snRNAs, 1,047 rRNAs, and 108 miRNAs (Fig. 6D). To validate the accuracy of small RNA predictions in the database, we performed miRNA sequencing on multiple tissue samples of *C. chinensis*. We obtained 60 miRNA sequences in *C. chinensis*, of which 19 overlapped with entries in the database, accounting for 17.59% of the total miRNAs recorded for *C. chinensis* in the database (Fig. 6D). For these overlapping miRNA sequences, we conducted human target gene prediction and KEGG pathway enrichment analysis (Fig. 6E). Our results indicated that the target genes of these miRNAs were enriched in pathways such as glycerolipid metabolism, gicotinate and nicotinamide metabolism, and efferocytosis. These pathways were potentially implicated in anti-atherosclerosis [63], anti-type 2 diabetes [64], anti-tumor [65], and anti-inflammatory [66] effects, aligning with the pharmacological applications of *C. chinensis*.

To further elucidate the regulatory roles of these miRNAs, we selected several representative miRNA-target gene-disease pathways from the KEGG enrichment results (Fig. 6F). We found that the *NUDT6* gene was closely associated with the regulation of cell proliferation and was regulated by six miRNAs: CM027378.1_4603, CM027380.1_11513, CM027383.1_21621, CM027383.1_21625, CM027383.1_21619, and CM027379.1_7695 [67]. In the efferocytosis pathway [68, 69], the *STYXL1* gene was linked to seminal vesicle tumor and epilepsy, [70] while the *UBXN11* gene in the protein processing in endoplasmic reticulum pathway was associated with thyroid hurtle cell adenoma and colon carcinoma in situ. [71] Both genes were regulated by the same miRNA, CM027379.1_9121. Additionally, the *PWWP2B* gene, also in the efferocytosis pathway and implicated in gastric tubular adenocarcinoma and sotos syndrome, was regulated by CM027381.1_15410. [72] The *AGPAT2* gene in the glycolipid metabolism pathway, which was associated with lipodystrophy, congenital generalized, type 1 and congenital generalized lipodystrophy, was regulated by CM027384.1_25862 [73].

### Mining of therapeutic potential of *Scolopendra subspinipes mutilans* small peptides

We employed *S. subspinipes mutilans* from the SHMC database as a model species for mining and predicting therapeutic potential of small peptides. Modern pharmacological studies have demonstrated that *S. subspinipes mutilans* exhibits notable bioactivities, including anti-tumor, anti-inflammatory, anti-bacterial, cardioprotective, and analgesic effects, largely attributed to its proteinaceous components [74, 75]. Previous proteotranscriptomic studies have identified centipede-derived polypeptide toxins, prompting us to further investigate its small peptides repertoire [76].

**Fig. 7:**
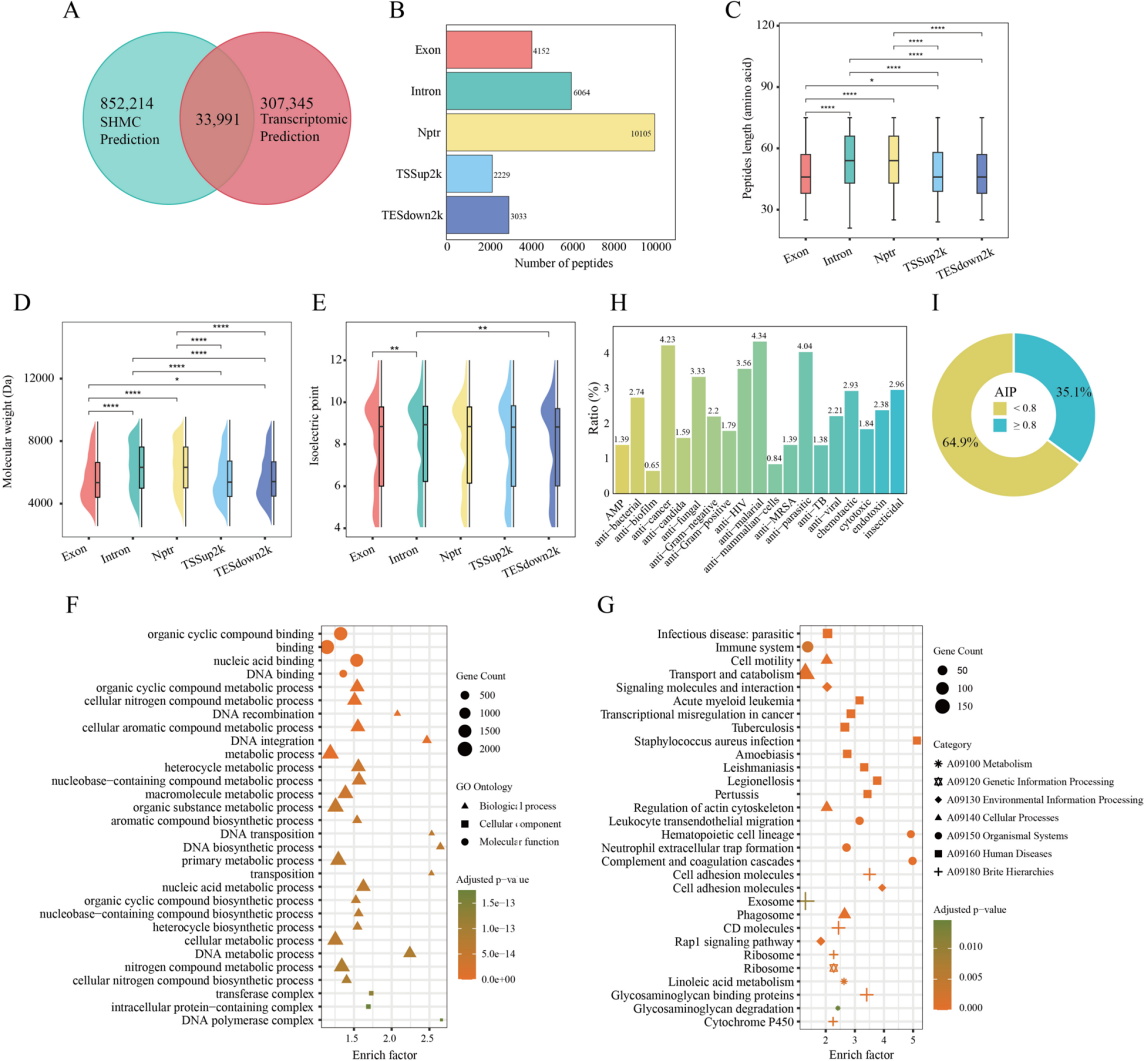
The integration analysis of SHMC prediction and sequencing of small peptides from *S. subspinipes mutilans*. **A** Venn diagram showing the number of small peptides identified by SHMC database and transcriptomic prediction, the number of overlaps represents validated small peptides. The areas shown in the diagram are not proportional to the number of peptides in each group. **B** Number of validated small peptides derived from different genomic feature regions. **C** Length of small peptides derived from different genomic feature regions. Boxes represent the second and third quartiles and whiskers represent 1.5 × IQR. **D** The half-violin plot combines box plot and kernel density trace to describe the molecular weight of the validated peptides derived from different genomic feature regions. **E** The half-violin plot describes the isoelectric point of the validated peptides derived from different genomic feature regions. **F** Bubble plot of GO enrichment analysis of small peptides that were completely located within different genomic regions. **G** Bubble plot of KEGG enrichment analysis of small peptides that were completely located within different genomic regions. **H** Histogram of predicted anti-microbial small peptides, the ratio represents the percentage of unique small peptides to the total number of small peptides under this type. **I** Pie chart of predicted anti-inflammatory small peptides (AIP). Probability index ≥ 0.8 was considered to have a credible anti-inflammatory effect. Wilcoxon rank-sum test was used for hypothesis testing, ^*^P < 0.05, ^**^P < 0.01, ^***^P < 0.001, ^****^P < 0.0001

By integrating 852,214 SHMC predicted sequences with 307,345 transcriptome derived small peptides, we identified 33,991 high confidence unique small peptides from *S. subspinipes mutilans* (Fig. 7A). Genome distribution of these peptides revealed diverse regions: 10,105 from Nptr completely, 4152 from exons, 6064 from introns, 2229 from transcription start site upstream 2 kb (TSSup2k) regions and 3033 from transcription end site downstream (TESdown2k) regions (Fig. 7B). This suggested that, in addition to canonical coding regions, Nptr in *S. subspinipes mutilans* exhibited significant translation activity. Length distribution analysis indicated that peptides derived from Nptr and intron regions were notably longer than those from other genomic features (Fig. 7C). Similarly, molecular weight analysis showed that Nptr- and intron-derived peptides were heavier, though their isoelectric points did not significantly differ across genomic regions (Fig. 7D, E).

To elucidate the potentially biological functions of these peptides, we performed GO and KEGG enrichment analyses. The GO terms highlighted enrichment in nucleic acid metabolism, organic matter metabolism and enzyme function (Fig. 7F), while KEGG pathways implicated these peptides in infection defense, immune regulation, and cancer-related processes (Fig. 7G). Anti-microbial function prediction revealed 19 distinct microbial defense mechanisms associated with *S. subspinipes mutilans* small peptides, underscoring their pharmaceutical potential (Fig. 7H). Through predictive screening, we further identified 8,336 unique AIP candidates, with 35.1% exhibited high-confidence anti-inflammatory activity (Probability index ≥ 0.8) (Fig. 7I). These results collectively validated the SHMC database as a powerful platform for systematic discovery of bioactive components from pan-Shennongjia medicinal species, facilitating omics-driven drug development through integrated computational analyses and experimental validations.

## Discussion

The remarkable species diversity and component specificity of pan-Shennongjia medicinal herbs have been profoundly shaped by the region’s unique eco-geological conditions over evolutionary timescales. Our study highlights two examples–*C. indicum* var. *aromaticum* and *D. flexicaule*–that have evolved distinct metabolic profiles characterized by abundant volatile compounds and polysaccharides, respectively. These traits reflect adaptive responses to regional high-altitude heterogeneous mountain system. Together, this illustrates a broader paradigm of environmentally driven components diversification in pan-Shennongjia species, which we conceptualize as a sequential molecular circuitry: “environmental pressure → genetic reprogramming → metabolic hyperaccumulation → ecological fitness optimization”. This evolutionary framework establishes pan-Shennongjia herbs as a model system for investigating environment-driven diversification of natural components and the emergence of medicinal properties through ecological adaptation.

The observed imbalance of component distribution for pan-Shennongjia herbs across phylogenetic tree revealed both biological and technical drivers of diversity. Biologically, the biosynthesis of many natural components is known to be lineage-specific and often follows phylogenetic signals. For instance, plant secondary metabolites frequently exhibit clade-dependent accumulation with clear phylogenetic signals [77, 78]. Technically, the uneven availability and quality of omics resources likely amplifies these differences. Species with well-assembled genomes, especially those with transcriptomic or proteomic data, tend to show richer peptide and sRNAs profiles, while those lacking genomic support often appear chemically sparse. These disparities highlight systemic structural imbalances in TCM research that reflect global patterns in traditional medicine research [79, 80]. A large proportion of components data is concentrated in a limited number of well-studied taxa, while many ecologically and culturally significant families, such as Crassulaceae and Polygonaceous, remain poorly characterized. This is particularly striking given that Crassulaceae is not only frequently used in the pan-Shennongjia region but is also statistically enriched compared to its representation in the ChP. Yet, the absence of compound records for most of its species reflects a critical knowledge gap that constrains our understanding of its therapeutic potential.

Given the exponential growth of multi-omics data, integrated omics-based approaches provide powerful tools for predicting, acquiring, and analyzing diverse natural components. These strategies could help address the existing imbalance and support the development of a more comprehensive and systematic research framework. Such efforts are essential for achieving a more inclusive and accurate representation of traditional medicinal taxa in critical ecotones like pan-Shennongjia, and for unlocking their full pharmacological promise. The SHMC database represents a fundamental step toward integrating diverse naturally occurring components within the framework of the central dogma of the molecular biology on a regional scale. This is also the most significant difference compared to existing TCM databases. The SHMC database extends beyond natural components themselves, demonstrating stronger regional characteristics in terms of herbal species coverage. Through multi-omics analysis, it achieves more comprehensive data composition in information acquisition. Functionally, it surpasses mere component analysis by incorporating target prediction, which significantly facilitates TCM based drug development research. For secondary metabolites and carbohydrates, their biosynthesis in living organisms is often controlled by complex pathways and regulatory networks, giving rise to diverse structures and functions. [81] Comparatively, small peptides and sRNAs components are directly encoded by the genome, where their two-dimensional sequences essentially determine three-dimensional structural characteristics. Therefore, potential component information can be maximally obtained through omics-based data analyses. However, the authentic biosynthesis of natural products involves remarkable complexity, and all four consequently component categories in SHMC may have limitations. Future efforts are required to expand the database and experimentally validate both identified secondary metabolites and carbohydrates as well as predicted small peptides and sRNAs.

Exploring the therapeutic potential of natural components from regional herbs is critical for drug discovery. In our study, *C. chinensis* serves as a prime example. With its diverse secondary metabolites, in particular the alkaloids, are long known for clinically relevant bioactivities. Based on the “component-target-pathway” network constructed using tools available in the SHMC database, we identified some components, such as oxyberberine, jatrorrhizine, and berberine, showing promising effects on anti-inflammation and stress responses. Compared with the extensive research on alkaloids in *C. chinensis*, the pharmacological potential of sRNAs in this herb remains largely unexplored. This study represents the first attempt to incorporate sRNAs into the investigation of *C. chinensis*’s therapeutic potential, revealing preliminary evidence that miRNAs may target human genes and regulate pathways such as glycerolipid metabolism and efferocytosis. Despite differences in their action modes, alkaloids and sRNAs may synergistically modulate disease pathways. Future research should integrate multi-omics data and experimental validation to elucidate the interactive effects of chemical constituents and sRNAs in *C. chinensis* and their regulatory networks involved in complex diseases.

The case of *S. subspinipes mutilans*, on the other side, highlights the pharmaceutical potential of small peptides. Our integrated genomic and transcriptomic analysis identified numerous high-confidence small peptides, predominantly originating from Nptr of the *S. subspinipes mutilans* genome. This was similar to those reported in plants of maize and *Arabidopsis*, revealing widespread transcriptional and translational activity in non-coding genomic regions [82]. Functional analysis of these centipede-derived peptides showed enrichment of pathways in relation to infection defense, immune regulation, and cancer-related processes, consistent with their predicted anti-microbial and anti-inflammatory properties [82]. Collectively, our work establishes a scalable framework for mining cryptic bioactive components across region-specific medicinal species, emphasizing the underutilized therapeutic value of natural components like sRNAs and small peptides in traditional medicine. In the near future, the implementation of genome-wide pan-GPCR drug screening platforms [83–85], combined with the mining of genome-wide natural products through databases such as SHMC, will drive a leapfrog development in drug discovery utilizing TCM and other regionally significant medicinal resources.

## Conclusions

Our analysis of 405 pan-Shennongja medicinal taxa revealed significant phylogenetic signals in both species’ diversity distribution patterns and their component accumulation profiles. These patterns underline the influence of regional environmental factors on adaptive evolution, as exemplified by our case studies of *C. indicum* var. *aromaticum* and *D. flexicaule*. To systematically characterize these pan-Shennongjia resources, we employed multi-omics data with artificial intelligence-assisted tools to develop the SHMC database, which captures diverse components and indicative information of their therapeutic potentials. Using SHMC, we selected two representatives of *C. chinensis* and *S. subspinipes mutilans* to experimentally validate the annotated components (e.g., sRNAs and small peptides) and computationally assess their therapeutic potential. In summary, this work established the first regional-scale, omics-based natural components database for central China, laying a foundation for future research, conservation, and therapeutic exploration of pan-Shennongjia’s endemic medicinal resources.

## Supplementary Information

Additional file 1

## Data Availability

The data in this study are available from the corresponding authors on reasonable request.

## Abbreviations

ADMET: Absorption, distribution, metabolism, excretion, and toxicity
AI: Artificial intelligence
AIP: Anti-inflammatory peptide
AMP: Anti-microbial peptide
ChP: The Pharmacopoeia of the People’s Republic of China
CSRF: Cross-Site Request Forgery
DFP: *D. huoshanense*’S polysaccharides
DHP: *D. huoshanense*’S polysaccharides
D-MPNN: Directed message-passing neural network
DOP: *D. officinale*’S polysaccharides
FoC: The Flora of China
Log P: Lipid-water partition coefficient
MEP: Methylerythritol phosphate
MNTD: Mean nearest taxon distance
MPD: Mean phylogenetic distance
MVA: Mevalonate
Nptr: Non-promoter-terminal regions
NRI: Net Relatedness Index
NTI: Nearest Taxon Index
ORM: Object-Relational Mapping
RAG: Retrieval-Augmented Generation
SHMC: Pan-Shennongjia Herbs Multi-Omics Components database
sRNAs: Small RNAs
TESdown2k: Transcription end site downstream 2 kb
THY-HCA: Thyroid hurtle cell adenoma
TPS: Terpene synthase
TSSup2k: Transcription start site upstream 2 kb
VOCs: Volatile organic compounds
