# Supplementary material for: Omics-based profiling and therapeutic potential of natural components in pan-Shennongjia medicinal herbs
Source: Chin Med. 2025 Oct 16;20:172. doi: 10.1186/s13020-025-01208-9 (PMC12529840; doi:10.1186/s13020-025-01208-9)
Supplement: Supplementary file 1 — Additional file 1 [file 13020_2025_1208_MOESM1_ESM.docx]

**Supporting Information**

**Table S1** Primer sequences for candidate terpene synthase (*TPS*) genes.

| **Primer name** | **Primer Sequence** |
| --- | --- |
| pET-CaroChr8G00073150-F | gcaaatgggtcgcggatccgaattcATGCCTTCTAAACAAGGA |
| pET-CaroChr8G00073150-R | tcgagtgcggccgcaagcttTCATATACTCATAGGATGA |
| pET-CaroChr6G00146020-F | gcaaatgggtcgcggatccgaattcATGTCTGTACAAGAGAATGTTA |
| pET-CaroChr6G00146020-R | tcgagtgcggccgcaagcttTCAGGATTGAGAATATTTAG |
| pET-CaroChr6G00145870-F | gcaaatgggtcgcggatccgaattcATGTCTGTACATGAGAGTGC |
| pET-CaroChr6G00145870-R | tcgagtgcggccgcaagcttTCATACCATCATAGGATCAAC |
| pET-CaroChr3G00011240-F | gcaaatgggtcgcggatccgaattcATGATGATTGCACAGAGCAT |
| pET-CaroChr3G00011240-R | tcgagtgcggccgcaagcttTTATGTGTCTTCTTTGGTTC |
| pET-CaroChr6G00145830-F | tcgagtgcggccgcaagcttATGTCTGTACAAGAGAAT |
| pET-CaroChr6G00145830-R | gcaaatgggtcgcggatccgaattcTCATACCATTATAGGATCAA |
| pET-CaroChr6G00136910-F | tcgagtgcggccgcaagcttATGGCTTTAATGTATTTCA |
| pET-CaroChr6G00136910-R | gcaaatgggtcgcggatccgaattcCTATTTCACCATTTGGATTG |
| pET-CaroChr1G00460010-F | tcgagtgcggccgcaagcttATGTCAACTTTATCTGTTTCT |
| pET-CaroChr1G00460010-R | gcaaatgggtcgcggatccgaattcTTAGACAATCATAGGGTGAAC |
| pET-CaroChr6G00138270-F | tcgagtgcggccgcaagcttATGGCCTTTACTGCGGATGA |
| pET-CaroChr6G00138270-R | tcgagtgcggccgcaagcttTCATATCTCTTGAATTGGAT |
| pET-CaroChr3G00011490-F | gcaaatgggtcgcggatccgaattcATGATGATTGCACAGAGCAT |
| pET-CaroChr3G00011490-R | tcgagtgcggccgcaagcttTTATGTGTGTTCTTTGGTTC |
| pET-CaroChr3G00011930-F | gcaaatgggtcgcggatccgaattcATGAAACCGGCCATCTTCTCT |
| pET-CaroChr3G00011930-R | tcgagtgcggccgcaagcttTTAGACTACAATTTCAAACA |
| pET-CaroChr3G00002490-F | gcaaatgggtcgcggatccgaattcATGTCTCTTAAAGAAGAAGAT |
| pET-CaroChr3G00002490-R | tcgagtgcggccgcaagcttTCATGTAATGATGCCATCAATA |
| pET-CaroChr6G00187080-F | tcgagtgcggccgcaagcttATGGCTTCAAAAGAAAATGAAAT |
| pET-CaroChr6G00187080-R | gcaaatgggtcgcggatccgaattcTCATATACTCATAGCATGAATGA |
| pET-CaroChr4G00263400-F | tcgagtgcggccgcaagcttATGGAGCATTCCTTGGCCT |
| pET-CaroChr4G00263400-R | gcaaatgggtcgcggatccgaattcTTAAATTTGACATAAACTATA |
| pET-CaroChr3G00021330-F | tcgagtgcggccgcaagcttATGGTCGACACACTTGAAAGATT |
| pET-CaroChr3G00021330-R | gcaaatgggtcgcggatccgaattcTTAATAAATCATTTCCTTCAC |
| pET-CaroChr8G00112230-F | tcgagtgcggccgcaagcttATGCATGAAGTATTTCATTACCG |
| pET-CaroChr8G00112230-R | tcgagtgcggccgcaagcttTTAAAAGCGCTCTTTCTCACTA |
| pET-CaroChr9G00280190-F | gcaaatgggtcgcggatccgaattcATGGAGATGCTTAAAGCTGATACT |
| pET-CaroChr9G00280190-R | tcgagtgcggccgcaagcttTTAATGTTGCATGGGGAT |
| pET-CaroChr3G00021320-F | gcaaatgggtcgcggatccgaattcATGATTGCACAGAGCATGTCGTCT |
| pET-CaroChr3G00021320-R | tcgagtgcggccgcaagcttTTATGTGTGTTCTTTGGTTCCATTC |
| pET-CaroChr5G00394470-F | gcaaatgggtcgcggatccgaattcATGTCCACTCTTCTAATTTGCAAT |
| pET-CaroChr5G00394470-R | tcgagtgcggccgcaagcttTTAAACTAAAGGATGAACAAGGAGCGA |
| pET-CaroChr6G00181500-F | tcgagtgcggccgcaagcttATGGCTCCACAACAAGAGGAAGT |
| pET-CaroChr6G00181500-R | gcaaatgggtcgcggatccgaattcTCAAATAGTCATAGGGTGA |
| pET-CaroChr1G00459730-F | tcgagtgcggccgcaagcttATGTCAACTTTATCTGTTTCTACT |
| pET-CaroChr1G00459730-R | gcaaatgggtcgcggatccgaattcTTAGAGAATCATAGGGTGA |
| pET-CaroChr3G00011460-F | tcgagtgcggccgcaagcttATGAAACCGGCCATCTTCTCT |
| pET-CaroChr3G00011460-R | gcaaatgggtcgcggatccgaattcTTAGACTACAATTTCAAACA |
| pET-CaroChr6G00186930-F | tcgagtgcggccgcaagcttATGGCTTCAAAAGAAAAT |
| pET-CaroChr6G00186930-R | tcgagtgcggccgcaagcttTCATATACTCATAGCATGAAC |
| pET-CaroChr9G00319130-F | gcaaatgggtcgcggatccgaattcATGAACAGTGCTCCATTGCCTTAT |
| pET-CaroChr9G00319130-R | tcgagtgcggccgcaagcttTCAAACACTCATAGCATGAACGA |
| pET-CaroChr8G00112240-F | gcaaatgggtcgcggatccgaattcATGGCCTCTAACGGGTTTTTGT |
| pET-CaroChr8G00112240-R | tcgagtgcggccgcaagcttTTAGCCCCTGGAGCTAGCTTTAA |
| pET-CaroChr9G00323850-F | gcaaatgggtcgcggatccgaattcATGTCAACTACTATTCCTCT |
| pET-CaroChr9G00323850-R | tcgagtgcggccgcaagcttTTAGATGATCATAGGGTGA |
| pET-CaroChr6G00151120-F | tcgagtgcggccgcaagcttATGCCTTCTAAACAAGGAGA |
| pET-CaroChr6G00151120-R | gcaaatgggtcgcggatccgaattcTCATAAACTCATAGGATGAATGA |
| pET-CaroChr2G00537940-F | tcgagtgcggccgcaagcttATGGCTTCAACATGCATTTCT |
| pET-CaroChr2G00537940-R | gcaaatgggtcgcggatccgaattcCTAGGATTTAGAATTGAAAAGTA |
| pET-CaroChr3G00038110-F | tcgagtgcggccgcaagcttATGAAGGAGATCGTTGAGA |
| pET-CaroChr3G00038110-R | gcaaatgggtcgcggatccgaattcTTATTTGAAATCTAAATGTGTAAT |
| pET-CaroChr9G00314570-F | tcgagtgcggccgcaagcttATGGCAACGGTTCAAGCTAATGT |
| pET-CaroChr9G00314570-R | tcgagtgcggccgcaagcttTTACGCGGGTAGAGAACCCACA |
| pET-CaroChr5G00353610-F | gcaaatgggtcgcggatccgaattcATGTCTACTAAGCAACAGGAAG |
| pET-CaroChr5G00353610-R | tcgagtgcggccgcaagcttTCATATGATGATAGCATCA |
| pET-CaroChr9G00322230-F | gcaaatgggtcgcggatccgaattcATGCCAGTCACTACGGTTGAACAACCAG |
| pET-CaroChr9G00322230-R | tcgagtgcggccgcaagcttCTATTCTATTTCTTGAATAGGATTGA |
| pET-CaroChr1G00459720-F | gcaaatgggtcgcggatccgaattcATGAGGGCATATCCGT |
| pET-CaroChr1G00459720-R | tcgagtgcggccgcaagcttTTAGAGAATCATAGGGTGAAC |

**Table S2** The NRI and NTI values calculated from phylocomr package under null model 1

|  | Pan-Shennongjia Herbs vs.  Chinese Pharmacopoeia Species | *P* values |
| --- | --- | --- |
| NRI values | -1.185318 | >0.05 |
| NTI values | -16.965977 | <0.001 |

**Table S3** 99 Upregulated terpenoids identified in *Chrysanthemum indicum* var. *aromaticum.*

| **Compounds** | **ID** |
| --- | --- |
| (1aR,1bS,2aS,5S,5aS,7aS)-2,2,5,7a-Tetramethyldecahydrocyclopenta[2',3']cyclobuta[1',2':3,4]benzo[1,2-b]oxirene | 358 |
| (1R,3aR,5aR,9aS)-1,4,4,7-Tetramethyl-1,2,3,3a,4,5a,8,9-octahydrocyclopenta[c]benzofuran | 191 |
| p-Mentha-1,8-dien-7-ol | 242 |
| (3S,3aR,3bR,4S,7R,7aR)-4-Isopropyl-3,7-dimethyloctahydro-1H-cyclopenta[1,3]cyclopropa[1,2]benzen-3-ol | 282 |
| .beta.-Oplopenone | 326 |
| (3E,7E)-1,5,5,8-Tetramethylcycloundeca-3,7-dienol | 310 |
| (3S,3aR,6R,8aS)-3,7,7-Trimethyl-8-methylenehexahydro-1H-3a,6-methanoazulen-2(3H)-one | 327 |
| (4R,4aR)-4,4a-Dimethyl-6-(prop-1-en-2-yl)-1,2,3,4,4a,7-hexahydronaphthalene | 95 |
| (5R,10R)-10-Methyl-6-methylene-2-(propan-2-ylidene)spiro[4.5]dec-7-ene | 328 |
| cis-.alpha.-Bisabolene | 329 |
| Naphthalene, decahydro-1,6-bis(methylene)-4-(1-methylethyl)-, (4.alpha.,4a.alpha.,8a.alpha.)- | 88 |
| (S,1Z,6Z)-8-Isopropyl-1-methyl-5-methylenecyclodeca-1,6-diene | 330 |
| 6-Methyl-2-(4-methylcyclohex-3-en-1-yl)hepta-1,5-dien-4-ol | 331 |
| 2-Methyl-6-(p-tolyl)hept-2-en-4-ol | 332 |
| 1,5-Heptadien-4-one, 3,3,6-trimethyl- | 173 |
| Furan, 3-(4-methyl-3-pentenyl)- | 182 |
| Cyclohexanone, 5-methyl-2-(1-methylethyl)- | 162 |
| cis-.beta.-Farnesene | 333 |
| Copaene | 52 |
| 2-Buten-1-one, 1-(2,6,6-trimethyl-1,3-cyclohexadien-1-yl)-, (E)- | 183 |
| (-)-.beta.-Bourbonene | 49 |
| trans-.alpha.-Bergamotene | 105 |
| (4aR-trans)-decahydro-4a-methyl-1-methylene-7-(1-methylethylidene)-Naphthalene | 119 |
| (E)-.beta.-Famesene | 334 |
| trans-.beta.-Ionone | 187 |
| Humulene | 335 |
| Germacrene D | 126 |
| (1S,2E,6E,10R)-3,7,11,11-Tetramethylbicyclo[8.1.0]undeca-2,6-diene | 106 |
| .alpha.-Farnesene | 336 |
| Cubenene | 1 |
| Benzene, 1-(1,5-dimethyl-4-hexenyl)-4-methyl- | 116 |
| Cyclohexene, 3-(1,5-dimethyl-4-hexenyl)-6-methylene-, [S-(R*,S*)]- | 124 |
| Naphthalene, 1,2,3,4-tetrahydro-1,6-dimethyl-4-(1-methylethyl)-, (1S-cis)- | 94 |
| 3-Buten-2-one, 4-(2,2,6-trimethyl-7-oxabicyclo[4.1.0]hept-1-yl)- | 209 |
| Caryophyllene oxide | 192 |
| Levomenol | 313 |
| .alpha.-Bisabolol | 312 |
| (1R,4R)-1-methyl-4-(6-Methylhept-5-en-2-yl)cyclohex-2-enol | 309 |
| 4,8-Methanoazulen-9-ol, decahydro-2,2,4,8-tetramethyl-, stereoisomer | 288 |
| 10-epi-.gamma.-Eudesmol | 307 |
| .alpha.-Calacorene | 70 |
| Neric Acid | 276 |
| Geranic acid | 275 |
| Bicyclo[3.1.1]hept-2-ene-2-carboxylic acid, 6,6-dimethyl- | 221 |
| 1-Cyclohexene-1-carboxylic acid, 4-(1-methylethenyl)- | 226 |
| (5R,6R)-3,6-Dimethyl-5-(prop-1-en-2-yl)-6-vinyl-6,7-dihydrobenzofuran-4(5H)-one | 215 |
| 2-Furanmethanol, tetrahydro-.alpha.,.alpha.,5-trimethyl-5-(4-methyl-3-cyclohexen-1-yl)-, [2S-[2.alpha.,5.beta.(R*)]]- | 322 |
| Santalol, E-cis,epi-.beta.- | 337 |
| 2H-Pyran, tetrahydro-4-methyl-2-(2-methyl-1-propenyl)- | 175 |
| (3aR,4R,7R)-1,4,9,9-Tetramethyl-3,4,5,6,7,8-hexahydro-2H-3a,7-methanoazulen-2-one | 189 |
| [1S-(1.alpha.,7.alpha.,8a.beta.)]-1,2,3,5,6,7,8,8a-octahydro-1,4-dimethyl-7-(1-methylethenyl)-Azulene | 103 |
| trans-Calamenene | 93 |
| Salvial-4(14)-en-1-one | 193 |
| .alpha.-Maaliene | 338 |
| Spiro[4.5]dec-7-ene, 1,8-dimethyl-4-(1-methylethenyl)-, [1S-(1.alpha.,4.beta.,5.alpha.)]- | 102 |
| Cedrol | 289 |
| Naphthalene, 1,2,4a,5,8,8a-hexahydro-4,7-dimethyl-1-(1-methylethyl)-, [1S-(1.alpha.,4a.beta.,8a.alpha.)]- | 81 |
| Naphthalene, 1,2,4a,5,6,8a-hexahydro-4,7-dimethyl-1-(1-methylethyl)-, [1S-(1.alpha.,4a.beta.,8a.alpha.)]- | 80 |
| (1R,4R,4aS,8aR)-4,7-Dimethyl-1-(prop-1-en-2-yl)-1,2,3,4,4a,5,6,8a-octahydronaphthalene | 90 |
| 1H-3a,7-Methanoazulene, 2,3,4,7,8,8a-hexahydro-3,6,8,8-tetramethyl-, [3R-(3.alpha.,3a.beta.,7.beta.,8a.alpha.)]- | 63 |
| 1-Methyl-4-(6-methylhept-5-en-2-yl)cyclohexa-1,3-diene | 339 |
| Epizonarene | 97 |
| (2R,3R,6S)-6-Isopropyl-3-methyl-2-(prop-1-en-2-yl)-3-vinylcyclohexanone | 194 |
| Cyclohexanol, 3-ethenyl-3-methyl-2-(1-methylethenyl)-6-(1-methylethyl)-, [1R-(1.alpha.,2.alpha.,3.beta.,6.alpha.)]- | 298 |
| (3S,3aS,6R,8aS)-3,8,8-Trimethyl-7-methyleneoctahydro-1H-3a,6-methanoazulene | 340 |
| 1H-Cycloprop[e]azulene, decahydro-1,1,4,7-tetramethyl-, [1aR-(1a.alpha.,4.beta.,4a.beta.,7.beta.,7a.beta.,7b.alpha.)]- | 56 |
| aR-Himachalene | 79 |
| Benzene, 1-methyl-4-(1,2,2-trimethylcyclopentyl)-, (R)- | 69 |
| 1,1,7,7a-Tetramethyl-1a,2,6,7,7a,7b-hexahydro-1H-cyclopropa[a]naphthalene | 341 |
| Bicyclo[4.4.0]dec-1-ene, 2-isopropyl-5-methyl-9-methylene- | 342 |
| 4,7-Methanoazulene, 1,2,3,4,5,6,7,8-octahydro-1,4,9,9-tetramethyl-, [1S-(1.alpha.,4.alpha.,7.alpha.)]- | 74 |
| Carotol | 305 |
| (4aR,8aR)-5,8a-dimethyl-3-propan-2-ylidene-1,2,4,4a,7,8-hexahydronaphthalene | 120 |
| (1R,3aS,8aS)-7-Isopropyl-1,4-dimethyl-1,2,3,3a,6,8a-hexahydroazulene | 343 |
| Azulene, 1,2,3,3a,4,5,6,7-octahydro-1,4-dimethyl-7-(1-methylethenyl)-, [1R-(1.alpha.,3a.beta.,4.alpha.,7.beta.)]- | 89 |
| (1S,4aR,7R)-1,4a-Dimethyl-7-(prop-1-en-2-yl)-1,2,3,4,4a,5,6,7-octahydronaphthalene | 344 |
| 1,4-Methanoazulen-9-ol, decahydro-1,5,5,8a-tetramethyl-, [1R-(1.alpha.,3a.beta.,4.alpha.,8a.beta.,9S*)]- | 287 |
| 1H-Naphtho[2,1-b]pyran, 3-ethenyldodecahydro-3,4a,7,7,10a-pentamethyl-, [3S-(3.alpha.,4a.alpha.,6a.beta.,10a.alpha.,10b.beta.)]- | 345 |
| 3H-3a,7-Methanoazulene, 2,4,5,6,7,8-hexahydro-1,4,9,9-tetramethyl-, [3aR-(3a.alpha.,4.beta.,7.alpha.)]- | 73 |
| 10,10-Dimethyl-2,6-dimethylenebicyclo[7.2.0]undecane | 346 |
| (-)-Aristolene | 64 |
| (1R,2R,4S,6S,7S,8S)-8-Isopropyl-1-methyl-3-methylenetricyclo[4.4.0.02,7]decan-4-ol | 347 |
| (1S,4S,4aR)-1-Isopropyl-4-methyl-7-methylene-1,2,3,4,4a,5,6,7-octahydronaphthalene | 348 |
| Valerena-4,7(11)-diene | 96 |
| Petasitene | 58 |
| 1,2,4-Methenoazulene, decahydro-1,5,5,8a-tetramethyl-, [1S-(1.alpha.,2.alpha.,3a.beta.,4.alpha.,8a.beta.,9R*)]- | 44 |
| 4-(1,5-dimethyl-1,4-hexadienyl)-1-methyl-Cyclohexene | 349 |
| (4R,4aS,6S)-4,4a-Dimethyl-6-(prop-1-en-2-yl)-1,2,3,4,4a,5,6,7-octahydronaphthalene | 350 |
| Eudesma-2,4,11-triene | 351 |
| (2R,8R,8aS)-8,8a-Dimethyl-2-(prop-1-en-2-yl)-1,2,3,7,8,8a-hexahydronaphthalene | 352 |
| (1S,4R,7S,8R,11R,13R)-4,7,11-Trimethyl-5-oxatetracyclo[5.4.2.01,8.04,13]tridecane | 353 |
| (-)-Spathulenol | 279 |
| Longifolenaldehyde | 354 |
| 2(3H)-Naphthalenone, 4,4a,5,6,7,8-hexahydro-4a,5-dimethyl-3-(1-methylethylidene)-, (4ar-cis)- | 196 |
| 1R,4R,7R,11R-1,3,4,7-Tetramethyltricyclo[5.3.1.0(4,11)]undec-2-ene | 355 |
| (1R,1aR,2aS,5R,6R,6aS,7aS)-1,6,6a-trimethyldecahydro-1,2a-methanocyclopropa[b]naphthalen-5-ol | 356 |
| Di-epi-.alpha.-cedrene-(I) | 357 |
| 1H-Cyclopropa[a]naphthalene, 1a,2,3,3a,4,5,6,7b-octahydro-1,1,3a,7-tetramethyl-, [1aR-(1a.alpha.,3a.alpha.,7b.alpha.)]- | 72 |
| Carvenone | 156 |

**Table S4** Biosynthetic advances in characteristic terpenoids of *Chrysanthemum indicum* var. *aromaticum.*

| **Compounds** | **CAS** | **Substrate** | **Reference** |
| --- | --- | --- | --- |
| p-Menthan-3-one,Cyclohexanone, 5-methyl-2-(1-methylethyl)- | 10458-14-7 | GPP | An *et al*. [1] |
| Perillyl alcohol,p-Mentha-1,8-dien-7-ol | 536-59-4 | GPP | Fujiwara *et al*. [2] |
| beta-Bourbonene，(-)-.beta.-Bourbonene | 5208-59-3 | FPP | Yao *et al*. [3] |
| Copaene | 3856-25-5 | FPP | Conart *et al*. [4] |
| Damascenone,2-Buten-1-one, 1-(2,6,6-trimethyl-1,3-cyclohexadien-1-yl)-, (E)- | 23726-93-4 | FPP | Wei *et al*. [5] |
| trans-.alpha.-Bergamotene | 13474-59-4 | FPP | Girolamo *et al*. [6] |
| Cyperene,3H-3a,7-Methanoazulene, 2,4,5,6,7,8-hexahydro-1,4,9,9-tetramethyl-, [3aR-(3a.alpha.,4.beta.,7.alpha.)]- | 2387-78-2 | FPP | Yu *et al*. [7] |
| Petasitene | 443124-67-2 | FPP | Zhou *et al*. [8] |
| beta-Maaliene,1H-Cyclopropa[a]naphthalene, 1a,2,3,3a,4,5,6,7b-octahydro-1,1,3a,7-tetramethyl-, [1aR-(1a.alpha.,3a.alpha.,7b.alpha.)]- | 489-29-2 | FPP | Liu *et al*. [9] |
| ALPHA-CEDRENE,1H-3a,7-Methanoazulene, 2,3,4,7,8,8a-hexahydro-3,6,8,8-tetramethyl-, [3R-(3.alpha.,3a.beta.,7.beta.,8a.alpha.)]- | 469-61-4 | FPP | Dickschat *et al*. [10] |
| Azulene, 1,2,3,3a,4,5,6,7-octahydro-1,4-dimethyl-7-(1-methylethenyl)-, [1R-(1.alpha.,3a.beta.,4.alpha.,7.beta.)]- | 22567-17-5 | FPP | Schmidt *et al*. [11] |
| Cubenene | 29837-12-5 | FPP | Chen *et al*. [12] |
| Levomenol | 23089-26-1 | FPP | Liu *et al*. [13] |
| Naphthalene, 1,2,4a,5,6,8a-hexahydro-4,7-dimethyl-1-(1-methylethyl)-, [1S-(1.alpha.,4a.beta.,8a.alpha.)]- | 24406-05-1 | FPP | Portnoy *et al*. [14] |
| Longifolenaldehyde | 19890-84-7 | FPP | Liu *et al*. [9] |
| 3,6,6,9-tetramethyl-1,4,4a,5,7,9a-hexahydrobenzo[7]annulene,(1S,2E,6E,10R)-3,7,11,11-Tetramethylbicyclo[8.1.0]undeca-2,6-diene | 24703-35-3 | FPP | Külheim *et al*. [15] |
| BETA-CADINENE | 523-47-7 | FPP | Kuo *et al*. [16] |
| .alpha.-Bisabolol | 515-69-5 | FPP | Zhou *et al*. [17] |
| Germacrene D | 23986-74-5 | FPP | Chen *et al*. [18] |
| Valerena-4,7(11)-diene | 351222-66-7 | FPP | Pyle *et al*. [19] |
| amorphadiene,(1R,4R,4aS,8aR)-4,7-Dimethyl-1-(prop-1-en-2-yl)-1,2,3,4,4a,5,6,8a-octahydronaphthalene | 92692-39-2 | FPP | Mercke *et al*. [20] |
| alpha-Bulnesene,[1S-(1.alpha.,7.alpha.,8a.beta.)]-1,2,3,5,6,7,8,8a-octahydro-1,4-dimethyl-7-(1-methylethenyl)-Azulene | 3691-11-0 | FPP | Kumeta *et al*. [21] |
| alpha-Bisabolene,4-(1,5-dimethyl-1,4-hexadienyl)-1-methyl-Cyclohexene | 17627-44-0 | FPP | Mafu *et al*. [22] |
| cubebol,(3S,3aR,3bR,4S,7R,7aR)-4-Isopropyl-3,7-dimethyloctahydro-1H-cyclopenta[1,3]cyclopropa[1,2]benzen-3-ol | 23445-02-5 | FPP | Jung *et al*. [23] |
| Cedrol | 77-53-2 | FPP | Luo *et al*. [24] |
| epi-Eudesmol,10-epi-.gamma.-Eudesmol | 15051-81-7 | FPP | Yu *et al*. [25] |

**Table S5** 40 alkaloid components in *Coptis chinensis.*

| **Compounds** | **CID** | **ID** |
| --- | --- | --- |
| Jatrorrhizine iodide | 5459338 | 1 |
| Palmatine Chloride | 73442 | 2 |
| Hydrastine | 197835 | 3 |
| (3S)-6,7-dimethoxy-3-(6-methyl-7,8-dihydro-5H-[1,3]dioxolo[4,5-g]isoquinolin-5-yl)-3H-2-benzofuran-1-one | 371942 | 4 |
| (+)-Hydrastine | 656743 | 5 |
| Chilenine | 11025386 | 6 |
| Jatrorrhizine Chloride | 371256 | 7 |
| 2,3,10-Trimethoxy-5,6-dihydroisoquinolino[2,1-b]isoquinolin-7-ium-9-ol;chloride | 10547385 | 8 |
| (7S,13aS)-2,3,10-trimethoxy-7-methyl-6,8,13,13a-tetrahydro-5H-isoquinolino[2,1-b]isoquinolin-7-ium-1,9-diol | 15432813 | 9 |
| Berberine Chloride | 12456 | 10 |
| Palmatine hydroxide | 200119 | 11 |
| Benzo(g)-1,3-benzodioxolo(5,6-a)quinolizinium, 5,6-dihydro-9-hydroxy-10-methoxy-, chloride | 72703 | 12 |
| (6As)-11-hydroxy-1,2,10-trimethoxy-6,6-dimethyl-5,6,6a,7-tetrahydro-4h-dibenzo[de,g]quinolinium | 161487 | 13 |
| Coptisine Chloride | 72321 | 14 |
| D-Tetrahydropalmatine | 969488 | 15 |
| 6H-Dibenzo[a,g]quinolizine, 5,8,13,13a-tetrahydro-2,3,9,10-tetramethoxy- | 5417 | 16 |
| Tetrahydropalmatine | 72301 | 17 |
| Pseudopalmatine | 644002 | 18 |
| Berberastine | 442180 | 19 |
| Oxyberberine | 11066 | 20 |
| Dihydrochelerythrine | 485077 | 21 |
| Jatrorrhizine | 72323 | 22 |
| Columbamine | 72310 | 23 |
| 16,17-Dimethoxy-6-tritio-5,7-dioxa-13-azoniapentacyclo[11.8.0.02,10.04,8.015,20]henicosa-1(13),2,4(8),9,14,16,18,20-octaene | 10246509 | 24 |
| Oxyberberrubine | 5384082 | 25 |
| Berberine | 2353 | 26 |
| Dehydroapocavidine | 9974201 | 27 |
| Epiberberine | 160876 | 28 |
| 8-Oxocoptisine | 5245667 | 29 |
| Corysamine | 147329 | 30 |
| Norisocorydine | 12313549 | 31 |
| Demethyleneberberine | 363209 | 32 |
| Groenlandicine | 3084708 | 33 |
| 17-Methoxy-5,7-dioxa-1-azoniapentacyclo[11.8.0.03,11.04,8.014,19]henicosa-1(13),2,4(8),9,11,14,16,18-octaen-16-ol | 45490416 | 34 |
| 16-Methoxy-5,7-dioxa-1-azapentacyclo[11.8.0.03,11.04,8.014,19]henicosa-2,4(8),9,11,13,15,18-heptaen-17-one | 11723579 | 35 |
| 1H-1,4-Benzodiazepine-7-carbonitrile, 2,3-dihydro-1-methyl-2-oxo-5-phenyl- | 19011 | 36 |
| 4-Hydroxy-3-methoxy-alpha-methylbenzyl Alcohol | 17203 | 37 |
| 4-(Hydrazinylmethyl)phenol | 17754229 | 38 |
| methyl (1R)-17-ethyl-3,13-diazapentacyclo[13.3.1.02,10.04,9.013,18]nonadeca-2(10),4,6,8,16-pentaene-1-carboxylate;hydrochloride | 72314 | 39 |
| Noroxyhydrastinine | 89047 | 40 |

**Supporting References**

1. An X, Liao Y, Yu Y, Fan J, Wan J, Wei Y, et al. Effects of MhMYB1 and MhMYB2 transcription factors on the monoterpenoid biosynthesis pathway in l-menthol chemotype of *Mentha haplocalyx* Briq. *Planta* 2024;**260**(1):3.

2. Fujiwara Y, Ito M. Molecular cloning and characterization of a *Perilla frutescens* cytochrome P450 enzyme that catalyzes the later steps of perillaldehyde biosynthesis. *Phytochemistry* 2017;**134**:26-37.

3. Yao S, Tan X, Huang D, Li L, Chen J, Ming R, et al. Integrated transcriptomics and metabolomics analysis provides insights into aromatic volatiles formation in *Cinnamomum cassia* bark at different harvesting times. *BMC Plant Biol* 2024;**24**(1):84.

4. Conart C, Simonsen HT. Tamariscol biosynthesis in *Frullania tamarisci*. *Phytochemistry* 2025;**229**:114301.

5. Wei Y, Wang Y, Meng X, Yao X, Xia N, Zhang H, et al. VviWRKY24 promotes β-damascenone biosynthesis by targeting *VviNCED1* to increase abscisic acid in grape berries. *Hortic Res* 2025;**12**(5):uhaf017.

6. Di Girolamo A, Durairaj J, van Houwelingen A, Verstappen F, Bosch D, Cankar K, et al. The santalene synthase from *Cinnamomum camphora*: reconstruction of a sesquiterpene synthase from a monoterpene synthase. *Arch Biochem Biophys* 2020;**695**:108647.

7. Yu N, Chen Z, Yang J, Li R, Zou W. Integrated transcriptomic and metabolomic analyses reveal regulation of terpene biosynthesis in the stems of *Sindora glabra*. *Tree Physiol* 2021;**41**(6):1087-102.

8. Zhou Z, Xian J, Wei W, Xu C, Yang J, Zhan R, et al. Volatile metabolic profiling and functional characterization of four terpene synthases reveal terpenoid diversity in different tissues of *Chrysanthemum indicum* L. *Phytochemistry* 2021;**185**:112687.

9. Liu Q, Peng J, Tao Z, Zhang J, Wu W, Tan Z, et al. Cloning and functional characterization of sesquiterpene synthase genes from *Inonotus obliquus* using a *Saccharomyces cerevisiae* expression system. *World J Microbiol Biotechnol* 2025;**41**(2):56.

10. Dickschat JS, Brock NL, Citron CA, Tudzynski B. Biosynthesis of sesquiterpenes by the fungus *Fusarium verticillioides*. *Chembiochem* 2011;**12**(13):2088-95.

11. Schmidt CO, Bouwmeester HJ, Bülow N, König WA. Isolation, characterization, and mechanistic studies of (-)-alpha-gurjunene synthase from *Solidago canadensis*. *Arch Biochem Biophys* 1999;**364**(2):167-77.

12. Chen C, Yao G, Wang F, Bao S, Wan X, Han P, et al. Identification of a (+)-cubenene synthase from filamentous fungi *Acremonium chrysogenum*. *Biochem Biophys Res Commun* 2023;**677**:119-25.

13. Liu D, Wang L, Gou L, Lu Y, Ma Y, Yao S, et al. Hybrid methylotrophic pathway in *Serratia marcescens* for sustainable terpenoid biosynthesis. *ACS Synth Biol* 2025;**14**(5):1766-76.

14. Portnoy V, Benyamini Y, Bar E, Harel-Beja R, Gepstein S, Giovannoni JJ, et al. The molecular and biochemical basis for varietal variation in sesquiterpene content in melon (*Cucumis melo* L.) rinds. *Plant Mol Biol* 2008;**66**(6):647-61.

15. Külheim C, Padovan A, Hefer C, Krause ST, Köllner TG, Myburg AA, et al. The Eucalyptus terpene synthase gene family. *BMC Genomics* 2015;**16**(1):450.

16. Kuo PM, Hsu KH, Lee, Yi R, Chu, Fang H, et al. Isolation and characterization of β-cadinene synthase cDNA from *Chamaecyparis formosensis* Matsum. *Holzforschung* 2012;**66**. 569-76.

17. Zhou F, Pichersky E. The complete functional characterisation of the terpene synthase family in tomato. *New Phytol* 2020;**226**(5):1341-60.

18. Chen H, Guo M, Dong S, Wu X, Zhang G, He L, et al. chromosome-scale genome assembly of *Artemisia argyi* reveals unbiased subgenome evolution and key contributions of gene duplication to volatile terpenoid diversity. *Plant Commun* 2023;**4**(3):100516.

19. Pyle BW, Tran HT, Pickel B, Haslam TM, Gao Z, MacNevin G, et al. Enzymatic synthesis of valerena-4,7(11)-diene by a unique sesquiterpene synthase from the valerian plant (*Valeriana officinalis*). *FEBS J* 2012;**279**(17):3136-46.

20. Mercke P, Bengtsson M, Bouwmeester HJ, Posthumus MA, Brodelius PE. Molecular cloning, expression, and characterization of amorpha-4,11-diene synthase, a key enzyme of artemisinin biosynthesis in *Artemisia annua* L. *Arch Biochem Biophys* 2000;**381**(2):173-80.

21. Kumeta Y, Ito M. Characterization of delta-guaiene synthases from cultured cells of Aquilaria, responsible for the formation of the sesquiterpenes in agarwood. *Plant Physiol* 2010;**154**(4):1998-2007.

22. Mafu S, Karunanithi PS, Palazzo TA, Harrod BL, Rodriguez SM, Mollhoff IN, et al. Biosynthesis of the microtubule-destabilizing diterpene pseudolaric acid B from golden larch involves an unusual diterpene synthase. *Proc Natl Acad Sci U S A* 2017;**114**(5):974-9.

23. Jung Y, Mitsuhashi T, Kikuchi T, Fujita M. Functional Plasticity of a viral terpene synthase, OILTS, that shows non-specific metal cofactor binding and metal-dependent biosynthesis. *Chemistry* 2024;**30**(31):e202304317.

24. Luo F, Ling Y, Li DS, Tang T, Liu YC, Liu Y, et al. Characterization of a sesquiterpene cyclase from the glandular trichomes of *Leucosceptrum canum* for sole production of cedrol in *Escherichia coli* and *Nicotiana benthamiana*. *Phytochemistry* 2019;**162**:121-8.

25. Yu F, Harada H, Yamasaki K, Okamoto S, Hirase S, Tanaka Y, et al. Isolation and functional characterization of a beta-eudesmol synthase, a new sesquiterpene synthase from *Zingiber zerumbet* Smith. *FEBS Lett* 2008;**582**(5):565-72.
